# Avoidance dynamics in tiger-leopard coexistence: a time-geographic approach

**DOI:** 10.1186/s40462-026-00681-7

**Published:** 2026-07-20

**Authors:** Yifei Liu, Somayeh Dodge, Achara Simcharoen, James L. D. Smith, Sean C. Ahearn

**Affiliations:** 1 Department of Geography, University of California Santa Barbara, Santa Barbara, California USA; 2 Department of National Parks, Wildlife and Plant Conservation, Nakhon Sawan, Thailand; 3 Department of Fisheries, Wildlife and Conservation Biology, University of Minnesota, Twin Cities, MN USA; 4 Hunter College – CUNY, New York City, NY USA

**Keywords:** Carnivore coexistence, Time geography, Null models, Delayed interaction, GPS

## Abstract

Large carnivores often share space yet face strong interference risks, raising a key question: when co-occurrence is observed, is their interaction intentional (attraction or avoidance) or incidental (random encounters)? Most overlap metrics blur this distinction and miss lagged or indirect interactions that may structure coexistence. We present a time-geographic framework to determine whether interactions are intentional or random, and at what temporal scales they occur, using a case study of tigers (*Panthera tigris*) and leopards (*Panthera pardus*) in Huai Kha Khaeng Wildlife Sanctuary in Thailand. Using rich long-term GPS tracking data from six tigers and five leopards with overlapping home ranges collected between 2017 and 2021, yielding 17 tiger-leopard dyads, we quantify interaction frequency and duration across sequential temporal partitions and test intentionality using a null model of correlated random walks. This approach reveals the dynamics of co-existence and competition between the two species. We further analyze these dynamics at the dyad level and in relation to prey distribution to assess how mapped large-bodied prey context mediates interspecific competition. Our results indicate that temporal staggering around prey patches, rather than pure spatial segregation, underpins tiger–leopard coexistence. Specifically, the analysis reveals two broader recurring interaction patterns: stable coexistence and home range shift competition, together with one rare high-conflict case. This framework separates intentional from incidental overlap, linking interaction regimes to prey landscapes and life history in multi-predator systems.

**Supplementary Information:**

The online version contains supplementary material available at https://doi.org/10.1186/s40462-026-00681-7.

## Introduction

Apex carnivores play vital roles in structuring ecosystems, but coexistence among them is often precarious when they compete for overlapping prey resources. Where body size and dominance differ markedly, subordinate species face elevated risks of interference competition and displacement, yet persistence alongside dominant predators is widespread in multi-carnivore systems [1–3]. In tropical Asia, tigers (*Panthera tigris*) and leopards (*Panthera pardus*) frequently share habitat, with substantial home range overlap and reliance on similar ungulate prey [2, 4]. For instance, in Huai Kha Khaeng Wildlife Sanctuary in Thailand, the two species coexist despite strong asymmetries, with leopards facing high interference risk in areas heavily used by tigers [4, 5]. Understanding whether leopards actively avoid tigers, or instead track them with delays to exploit prey killed by tigers, is central to understanding coexistence strategies and anticipating conservation outcomes.

Coexistence is often attributed to spatial segregation, temporal partitioning, or prey specialization [1, 6, 7]. Yet most studies reduce interactions to static home range or activity overlap [8, 9]. The lack of temporal dynamics makes it impossible to determine whether co-occurrence reflects intentional avoidance or following behavior, rather than incidental or random encounters due to shared habitats. Proximity thresholds and step-selection models capture short-term attraction and avoidance [10, 11], but remain sensitive to sampling design and often miss asynchronous interactions spanning hours to days to weeks [12, 13]. These limitations make it difficult to diagnose the behavioral mechanisms that underpin coexistence or conflict, particularly in systems where interactions unfold across multiple temporal scales or shift with life history and prey [14].

Recent advances in movement ecology, driven by the proliferation of long-term and high-resolution animal tracking data, provide an opportunity to close this gap [15, 16]. Time-geographic methods model feasible space use under motion capacities and speed–time constraints, enabling detection of both concurrent (synchronous) and delayed (asynchronous) interactions [12, 13, 17]. Concurrent encounters elevate conflict risk, while temporally separate visits may reduce potential conflict and allow sequential exploitation of live prey and carcasses or cautious tracking of a dominant competitor [2, 6]. Coupled with null models that generate baseline expectations from randomized trajectories [18, 19], temporal analyses allow researchers to distinguish intentional encounters from chance co-occurrence. Our contribution is to extend this approach by integrating external and internal factors (e.g. gender, life stage, prey occupancy) with partitioned time-geographic analysis, while employing correlated random walk null models of movement within a Monte Carlo Simulation to evaluate the intention and nature of interaction among animals. Thus, we capture how intentionality shifts through time and link behavioral signals to ecological context. This approach directly addresses the challenge of non-stationary interaction regimes, where strategies may transition from coexistence to displacement as mapped large-bodied prey context or life history changes.

The proposed methodology is evaluated through a case study aiming to determine whether, when, and at what temporal scales leopards intentionally avoid or follow tigers within shared home ranges in Huai Kha Khaeng Wildlife Sanctuary. Specifically, we (1) quantify tiger-leopard interactions across sequential temporal partitions of GPS tracking data (i.e. approximately monthly time partitions); (2) test intentionality versus random overlap by comparing observed concurrent durations and directional following event frequencies against a partition-specific null model; and (3) evaluate how prey distribution (Sambar, Banteng, Gaur) influences spatial overlap and temporal interaction strategies between tigers and leopards of different age and gender. By linking interaction regimes to prey landscapes and individual life history, we identify conditions associated with stable coexistence, reduced overlap, and one rare high-conflict case.

## Background

### Interaction analysis

Research on predator coexistence has long quantified spatial and temporal overlap. Spatially, utilization distribution models (UDs) from kernel density estimators remain the standard for measuring home range overlap, with indices such as Bhattacharyya’s affinity and volume of intersection capturing both extent and intensity of shared space use [8, 9, 20]. Temporally, overlap is often estimated from camera traps or GPS timestamps, with kernel methods on circular time providing metrics of diel activity overlap [21–23]. These approaches show that species ranges and activity windows frequently coincide, but they cannot reveal whether co-occurrence reflects behavioral responses or incidental overlap.

To move beyond static overlap, movement-based approaches assess dynamic interactions along trajectories. Proximity and co-occurrence indices quantify time spent within thresholds of distance and duration [11, 24–26], while step-selection functions (SSFs) incorporate conspecific or heterospecific locations as covariates, testing whether individuals move toward, away from, or independently of others [10, 27, 28]. These methods detect fine-scale attraction and avoidance. However, they are sensitive to sampling resolution and thresholds, and often miss lagged interactions, which can be ecologically meaningful in systems with coarse telemetry or asynchronous prey use.

Time geography offers an alternative by explicitly modeling feasible movement under speed–time constraints. Originating in human mobility studies [29, 30], it represents individuals’ Potential Path Areas (PPAs) that delimit where an animal could plausibly be given movement capacities and time [31–33]. Adaptations for wildlife now detect both concurrent encounters and delayed interactions [34, 35]. ORTEGA (Object- oRiented TimE-Geographic Analytical approach) extends this line by treating PPAs as computational objects, enabling efficient large-scale intersection of asynchronous trajectories [12, 13, 36]. Compared to kernel overlap or proximity thresholds, time-geographic methods are more robust to variation in sampling resolution and can capture intentional lagged following that other approaches often miss [37]. Yet, despite recent applications to large carnivores [14], they remain rarely applied in carnivore ecology, leaving open questions about how intentionality signals link to prey landscapes, dispersal, and territorial change.

### Intentionality assessment

Spatial overlap alone does not imply intentionality, as individuals may co-occur incidentally due to shared habitat or resources. Intentionality implies systematic attraction (e.g., following at specific lags) or repulsion (avoidance) beyond random movement and habitat preferences. Disentangling intentional from incidental overlap is critical for identifying mechanisms such as risk avoidance, scavenging at kills and for management decisions aimed at mitigating interference and displacement.

Null models are a common strategy to distinguish intentional behavior from random patterns. By preserving individual movement properties (step lengths, turning angles, autocorrelation) while disrupting inter-individual synchrony, they generate expected baselines of incidental overlap [18, 19]. Approaches include trajectory permutation or rotation [10], time or space shifts [19], or step-selection models that embed conspecific or heterospecific positions as covariates to test attraction/avoidance [28, 38]. Hidden Markov and state–space models extend this further by identifying interaction states from switching behaviors [39].

Despite these advances, most applications evaluate single scales or compute trajectory-wide overlap, which obscures life-history transitions and lag-structured following [19, 40]. Proximity metrics also remain threshold-dependent [11]. Time-geographic approaches show promise for detecting delayed interaction [12, 35], but have rarely been combined with time partitioned null model testing to capture nonstationary interaction regimes. Closing this gap requires methods that integrate lag-explicit null tests with temporal windows to reveal when and at what lags animals intentionally co-locate or avoid one another. To address these limitations, we introduce two complementary indices that compare interaction intensity relative to home range overlap and summarize both the direction and magnitude of interaction patterns across temporal partitions.

## Methods

**Fig. 1:**
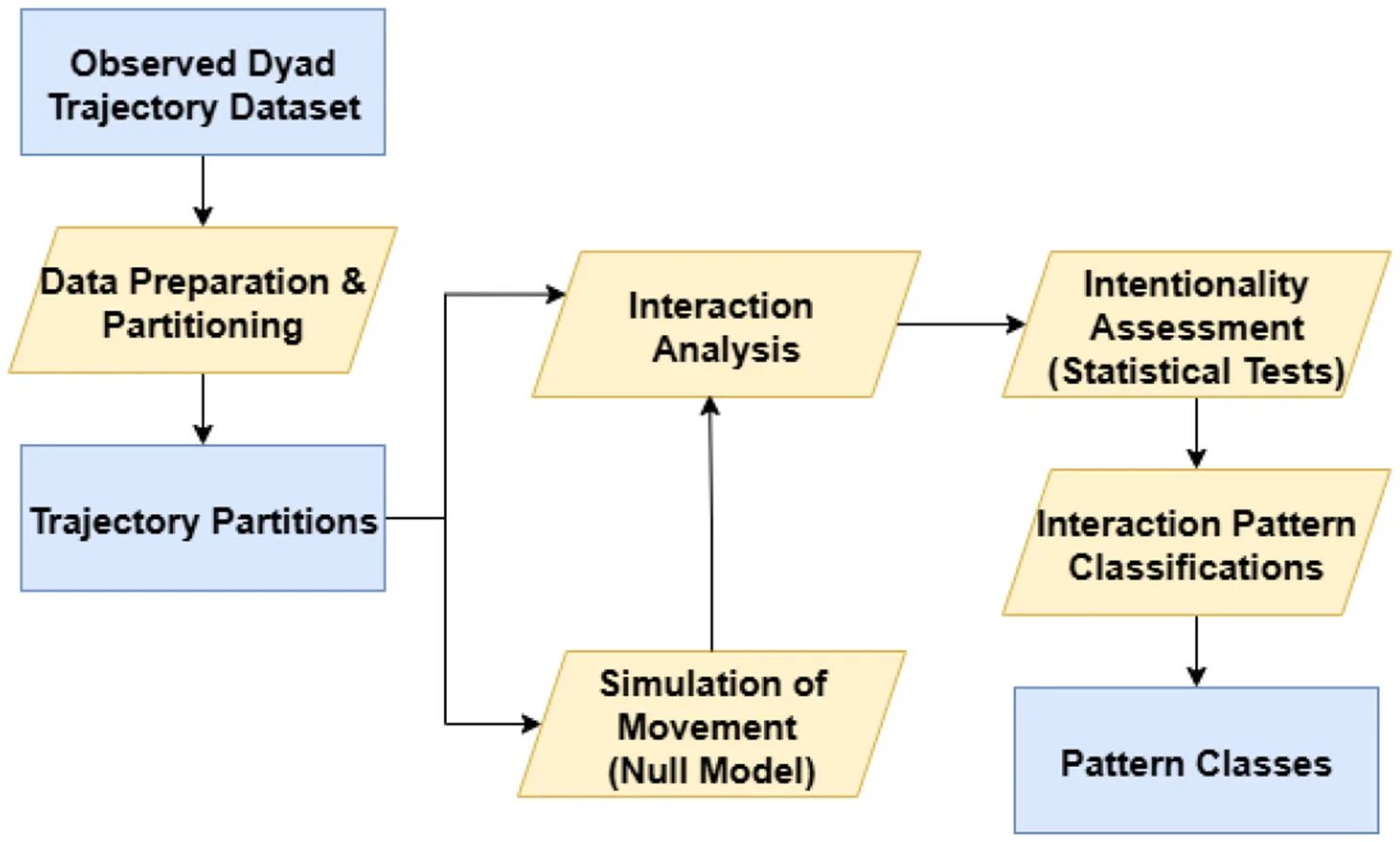
Analytical workflow for detecting and classifying animal interaction patterns

Figure 1 summarizes the methodological workflow, which involves the following components: (1) data preparation and temporal partitioning, (2) interaction analysis, (3) null model simulation, (4) statistical tests for comparison between observed interactions and null models, (5) interpretation and classification of interaction patterns. In this section, we first introduce the case study, followed by a detailed description of the methodology.

### Study area and data

Our core study area is Huai Kha Khaeng (HKK) Wildlife Sanctuary in Thailand, a protected area of 2,780 km$^2$ for Indochinese tigers and leopards. We use movement observations from GPS tracking of six tigers and five leopards. The tracking data were collected between 2017 and 2021 at 15–60 min intervals, with individual trajectories spanning 4–28 months. For illustrative purposes, here, we highlight three dyadic case studies using two tigers (FT1, MT3) and two leopards (ML2, ML4), which provided long-duration, high-resolution data (Table 3). We provide the data and analytical outcomes of other dyads in the supplementary materials (see Appendix A).

Environmental covariates describing prey distribution are incorporated as input layers in the analysis. Mapped large-bodied prey context is quantified using occupancy models for Sambar (*Rusa unicolor*), Gaur (*Bos gaurus*), and Banteng (*Bos javanicus*) from Jornburom et al. [41]. Following Jornburom et al. [41], occupancy values * > 0.6* are considered ecologically meaningful for supporting large carnivore presence [41].

### Data preparation and temporal partitioning

Prior to analysis, all trajectories are pre-processed to ensure data quality. Records with missing or invalid coordinates or different locations associated with duplicated timestamps are removed. For each individual, we then compute step lengths, time intervals, and speed values between successive GPS fixes. Step lengths exceeding the maximum movement capacity of tigers and leopards (6 km/h, $99.9^{th}$ percentile of observed steps) are removed, as are biologically implausible positions resulting from GPS error.

For each tiger-leopard pair, we restrict analysis to the period when both collars are simultaneously active, discarding fixes outside the shared tracking interval. To capture how interactions change over time and for computational efficiency, the shared interval of the tracking data is then partitioned into consecutive 1,000 hour (h) “partitions”. This temporal segmentation allows us to track shifts in interaction patterns across comparable time blocks. The choice of 1,000 h (*≈* 41 days) balances frequency of the observations with ecological relevance, allowing detection of temporal behavioral shifts while preserving sufficient data density. We evaluate the robustness of this partition length by repeating the analysis for the three pairs using 500 h and 2,000 h partitions (see Appendix B).

### Interaction analysis

#### Quantify interaction events and duration

We use a time-geographic approach to quantify tiger-leopard interactions [12]. Specifically, we use the ORTEGA framework, developed by Su et al. [13], to apply interaction analysis between each pair for each time partition. ORTEGA models movement of each individual between observation points as a set of potential path areas (PPAs). The PPA delimits the set of locations accessible by the individual given its speed capacity and the time budget between two consecutive observations. Intersections of PPAs along the trajectories of the two individuals identify opportunities (or potential areas) for their interaction. This spatiotemporal relationship can be formally expressed as [12, 35, 36]:

1 $$\begin{aligned}&PPA_{j,j+1}^{B} \cap PPA_{i,i+1}^{A} \\ &= \begin{cases}\varnothing, & \text{no interaction},\\\neq \varnothing \ \mathrm{and}\ [t_j, t_{j+1}] \cap [t_i, t_{i+1}] \neq \varnothing, & \mathrm{concurrent},\\\neq \varnothing \ \mathrm{and}\ [t_j, t_{j+1}] \cap [t_i - T,\, t_{i+1} + T] \neq \varnothing, & \mathrm{delayed.}\end{cases}\end{aligned}$$

Here, the intersection of PPAs $(PPA_{j,j+1}^{B} \cap PPA_{i,i+1}^{A})$ identifies spatial overlap, while the temporal intersection terms $[t_j, t_{j+1}]$ and $[t_i, t_{i+1}]$ determine whether two individuals are simultaneously present and move together (concurrent) or they visit the same location with a temporal lag *T* (delayed).

To investigate encounters (concurrent interaction) and following behaviors (delayed interaction) among tigers and leopards, we quantify interaction frequencies and duration across seven progressively increasing lag bins (0–1 h, 1 h-1 day(d), 1–2 d, 2–4 d, 4–8 d, 8–16 d, 16–21 d). Short lags (*≤ 1 d*) capture direct encounters or short-term avoidance following shared space use, while multi-day lags (* > 1 d*) reflect delayed revisits potentially related to foraging, scavenging, or scent-mark tracking. We consider lags up to three weeks because tiger scent marks can persist and remain detectable for this duration [42].

For each partition and time lag bin, we first use ORTEGA to extract all continuous spatiotemporal overlap segments between tiger and leopard PPAs, then compute two observed metrics: (1) total duration of concurrent interaction and (2) the number of tiger- vs. leopard-following events. The total concurrent interaction duration is the sum of the duration of all interaction events within that partition. To determine the direction of delayed co-location, we compare each individual’s first entry into the overlapping area: if the start time of tiger’s PPA is earlier than leopard’s within a delayed interaction sequence, we record a leopard-following event, and vice versa. These per-partition, per-lag summaries serve as the empirical benchmarks for subsequent null-model comparisons (as described in Section “Null model simulation of independent movement”).

#### Quantify interaction strength

Each PPA intersection sequence indicates a potential interaction event, but does not provide a global estimate of interaction strength (weak or strong) and types of interactions (avoidance or colocation). To characterize these dimensions, we introduce two complementary indices to better classify interaction type and quantify its overall strength given the home range area overlap between two animals: (1) Relative interaction intensity *R*, and (2) composite magnitude of interaction *M*.

For each partition, let *P* denote the proportion of intersected PPAs, defined as the number of intersected PPAs divided by the total number of PPAs across both individuals. Let *A* denote the overlapped home range area (km$^2$). We define the interaction density *D* in the shared home range area as:

2 $$ D = \frac{P}{A}$$

where high *D* values indicate more interaction opportunities per unit of shared space. Partitions with *A=0* are not considered, as no home range overlap implies no possibility of interaction.

The mean interaction density across all partitions of each pair $\bar{D}$ provides a baseline expectation. The *relative interaction intensity index* *R* compares each partition’s interaction density to this baseline:

3 $$ R = \frac{D-\bar{D}}{D+\bar{D}} \qquad \in [-1, 1]$$

If *R ≈ +1*, it means much higher than average interaction density given small overlap area, trending to exhibiting a *colocation* behavior during that partition. If *R ≈ -1* means much lower than average interaction density given a large overlap area, trending to exhibiting an *avoidance* behavior in that partition. The midpoint (*R ≈ 0*) means that the observed interaction density matches the average and no significant interaction behavior can be inferred.

While *R* captures the relative density of interaction, it overlooks the absolute scale of shared space, especially in cases where *R ≈ 0*. To better distinguish between partitions that have high interaction density over large overlap area versus low interaction density over small overlap area, we introduce a complementary magnitude index. Specifically, we log-transform and standardize both *P* and *A* to obtain z-scores ($z_{P}$ and $z_{A}$). The z-scores are calculated within each dyad across all valid temporal partitions with non-zero home-range overlap, so that *M* describes the relative magnitude of interaction within the same pair rather than differences in home-range size among pairs. Their sum gives a *composite interaction magnitude score*:

4 $$ M = z_{P} + z_{A}$$

We then rescale *M* to the range *[-1,1]* for comparability. Here, high values (*M ≈ +1*) indicate a *strong* interaction pattern when a large proportion of intersected PPAs are observed in a large overlap area. Low values (*M ≈ -1*) indicate a *weak* interaction when a small proportion of intersected PPAs are observed in relatively small overlap area of home ranges.

Together, *R* (relative intensity: avoidance vs. colocation) and *M* (composite magnitude: weak vs. strong) form a two-dimensional interaction space. Each partition can be placed within one of four quadrants. These indices provide a standardized framework for comparing interaction patterns across dyads and partitions. We provide a schematic quadrant diagram in Appendix Figure C3 to illustrate this interpretation.

### Null model simulation of independent movement

We apply a null model simulation approach [18, 19] to assess the significance of observed interaction patterns and provide statistical confirmation of intentional behaviors. Null models are widely used in ecology to compare observed data with patterns generated under random conditions [18]. The process involves: (1) selecting a statistic or an index that quantifies the pattern of interest, (2) generating a distribution under the null hypothesis with simulated values, and (3) comparing the observed statistic to assess significance [19]. This approach will be applied to analyze whether observed interactions are due to intentional behaviors, particularly for patterns like “avoidance”.

As the null model for each partition, we simulate independent Correlated Random Walks (CRWs) [43]. The CRW simulates each individual independently, which preserves individual movement characteristics but removes any behavioral dependence between them. Step lengths and turning angles are sampled from empirical distributions fitted to the entire trajectory of each individual (e.g., log-normal for step length; von Mises for turning angles), and simulations match observed timestamps to preserve temporal autocorrelation.

Each simulated path is constrained spatially within the extent of the home range boundary for the time partition. The home range is computed as the minimum convex polygon of the observed points within each partition [44]. To avoid unrealistic movement into unsuitable terrain, we impose a slope threshold, excluding locations exceeding the maximum slope value observed in the entire trajectory [45]. Starting positions and initial bearings are randomized, and any steps leaving the feasible domain are redrawn. This procedure maintains observed movement autocorrelation while enforcing realistic boundaries.

We generate up to 1,000 Monte Carlo replicates per partition, yielding ensembles of simulated trajectories at the same temporal resolution as the observed data. These ensembles are then subjected to the same interaction analysis (described in Section “Interaction analysis”) as the observed trajectories, producing null distributions for concurrent interaction durations and tiger-/leopard-following frequencies.

### Assessing interaction intentionality

To test whether observed interactions deviate from random expectation, we compare partition – and lag–specific concurrent durations and tiger-/leopard-following event counts to null distributions from the Monte Carlo CRW simulations using two-sided Mann–Whitney U tests. To reduce Type I error inflation from repeated pair-partition-lag comparisons, we use a conservative significance threshold of *α = 0.01* for assigning local interaction states. We also evaluate robustness using false-discovery-rate correction as a supplementary sensitivity check (see Appendix D) [46, 47].

For each partition and time lag bin, we test the null hypothesis $H_0$ that the simulated total duration and the observed duration arise from the same distribution, while the alternative hypothesis is that their medians differ. The Mann-Whitney statistic *U* compares the observed value against the 1,000 simulated values. The resulting two-sided *p*-value is computed exactly, and we declare the duration in specific partition and time lag bin to be significantly different from the null at *α = 0.01* if *p < 0.01.* A significant result indicates intentional concurrent interactions, while *p ≥ 0.01* implies that the observed concurrent interactions within that partition can be regarded as random encounters.

We perform another Mann-Whitney U test on interaction frequency broken down by tiger-/leopard following of observed and simulated tracks. For each following direction, partition, and time lag bin, we test the null hypothesis $H_0$ that the simulated interaction frequency and the observed frequency arise from the same distribution, while the alternative hypothesis is that their medians differ. Similarly, a two sided Mann-Whitney U test is conducted, and significance is declared when *p < 0.01.* A significant result thus indicates that the observed directional following frequency differs from the null expectation, revealing potential awareness, avoidance, or structured following behavior between the tiger and leopard when interpreted together with the interaction indices (*R* and *M*).

### Classification of interaction patterns

We classify interaction patterns at two hierarchical levels: (1) *local short-term patterns*, which capture the short-term behavioral context of interaction within each 1,000-hour window and across time lags, and (2) *global long-term dynamics*, which summarize the overall behavioral nature of a tiger-leopard dyad through time.

#### Local short-term patterns

Each partition–lag combination is assigned to one of three core interaction states: *Encounter*, *Following*, or *Avoidance*, based on the significance tests and interaction indices (*R* and *M*) defined in Section “Quantify interaction strength”. Here, *Encounter* represents concurrent colocation (0–1 h), and *Following* denotes delayed sequential use (1 h–21 d), expressed as tiger-following or leopard-following depending on direction. Together, they constitute two temporal forms of *Colocation* as defined in Section “Quantify interaction strength”. These three states provide behavioral context for interpreting the temporal and spatial nature of interaction (Table 1). Two additional auxiliary conditions are recorded but not treated as independent states: *no overlap*, denotes lack of spatial overlap (*A=0*), and *bidirectional following*, marking partitions with both concurrent and bidirectional following significance.

**Table 1:** Partition-level interaction states and their ecological interpretation. Each state represents the behavioral context of interaction within a partition across time lags

Pattern	Definition	**Criteria (***R*, *M***)**	Ecological meaning
Encounter	Frequent significant concurrent interaction (*0-1 h*)	*R ≥ 0*, *M ≈ 0*	Intense interaction or potential conflict in a shared area
Following	Significant delayed interaction (*1 h-21 d*), where one species consistently follows the other’s prior locations	*R ≥ 0*, *M > 0*	Sequentially tracking of the other’s movement, e.g., scavenging or foraging shared prey
Avoidance	No significant interaction while having a high spatial overlap	*R < 0*, *M ≤ 0*	Spatiotemporal separation or active avoidance to reduce conflict

Because each partition contains multiple lag-specific tests, the assigned local state represents an expert-informed dominant summary rather than the output of a fully automated decision rule. For sparse or mixed-evidence partitions, the label should be interpreted as a dominant interaction tendency rather than definitive evidence of behavioral motivation.

#### Global long-term dynamics

To characterize the overall interaction dynamics of the 17 tiger–leopard pairs, we summarize each pair’s sequence of local-level short term patterns across its entire trajectory using a supervised classifier. For each pair, we compute the proportion of partitions labeled as *tiger-following*, *leopard-following*, *avoidance*, *no overlap*, and *bidirectional following*, together with the number of non-empty partitions. From these proportions we derive a *follow-balance* index (tiger-following minus leopard-following) and an *avoidance-dominance* index (avoidance minus the maximum of tiger- or leopard-following). We also include a binary indicator for *bidirectional following*, indicating that both tiger-following and leopard-following are significant within at least one partition, often accompanied by frequent encounters. We fit a multinomial logistic regression classifier (one-vs-rest), with standardized predictors, to assign each pair to one of three pair-level categories: two broader recurring interaction patterns and one rare high-conflict category (Table 2). Reference labels come from a priori ecological classification (high-conflict dynamics, stable coexistence, or home range shift competition) based on joint inspection of partition-level patterns, trajectories, and prey occupancy distributions. Leave-one-out cross-validation yields 94.1% agreement between model predictions and these expert labels. The sole mismatch is the only pair that ever exhibits *bidirectional following*. Motivated by this rare but ecologically meaningful pattern, we apply a simple rule-based override: if any partition is labeled *bidirectional following*, the pair is classified as *High-conflict dynamics*. This knowledge-guided rule corrects the single mismatch and yields 100% agreement with the expert labels. We interpret this agreement as consistency with the expert-informed labels rather than independent proof of predictive performance. These pairwise dynamics represent long-term ecological outcomes emerging from repeated short-term interactions. The leave-one-dyad-out predictions and confusion matrices are provided in Appendix Section E.

**Table 2:** Pairwise interaction dynamics summarizing the overall interaction nature of a tiger–leopard dyad over time. High-conflict dynamics is represented by one rare dyad, whereas stable coexistence and home range shift competition represent broader recurring patterns

Type	Dominant pattern	Ecological meaning
High-conflict dynamics	Frequent encounters, bidirectional following	Escalated interaction and competition for shared space or prey
Stable coexistence	Following dominates with sustained home range overlap	Temporal partitioning of shared prey patches without direct confrontation
Home range shift competition	Avoidance dominates, often leading to disappearance of overlap over time	Spatial displacement and competitive exclusion

## Results

### Overall description

**Fig. 2:**
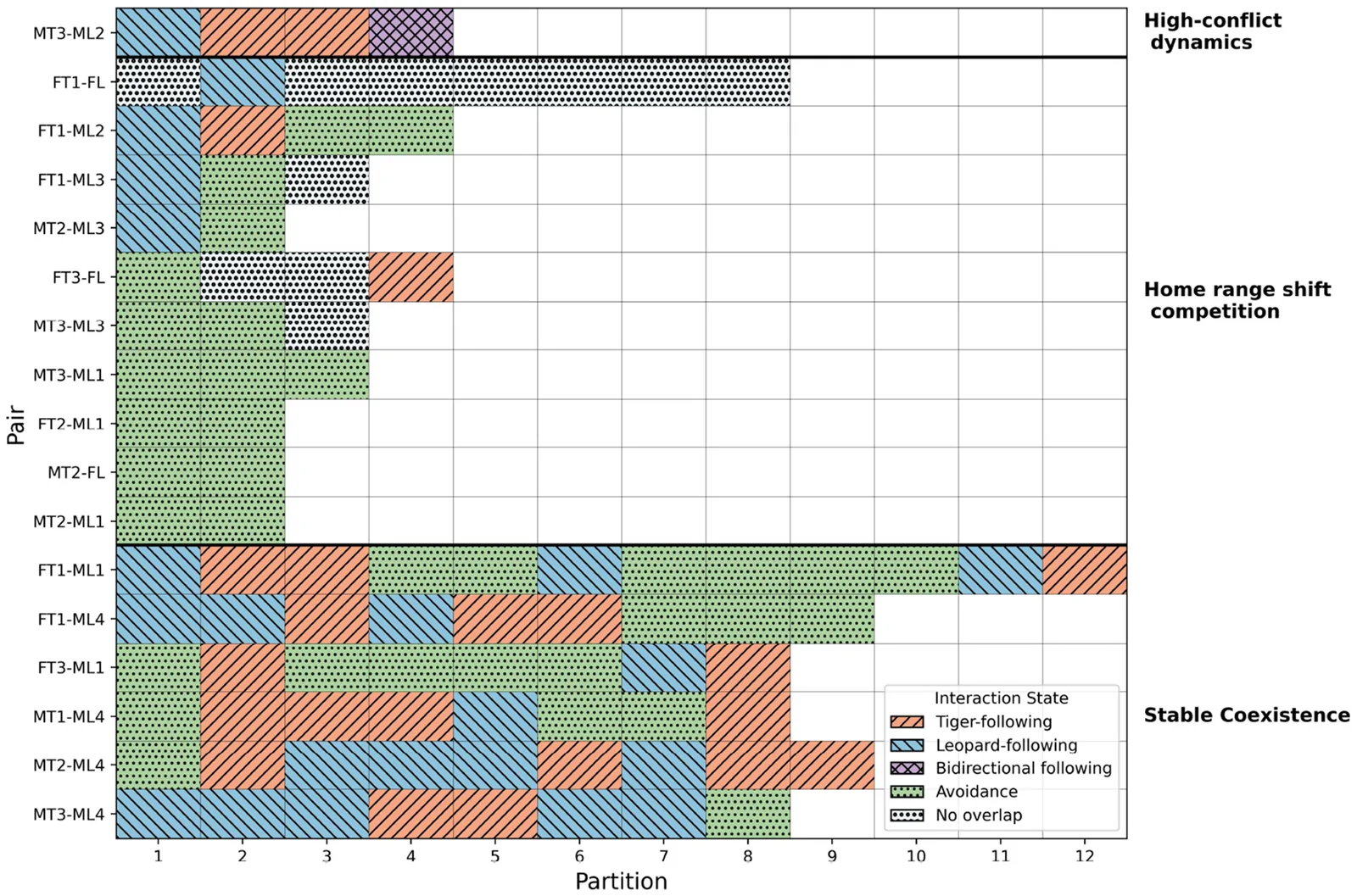
Interaction patterns between tiger-leopard pairs across sequential time partitions. Rows are pairs and columns are partitions. Each cell is filled with a color-hatch combination corresponding to the classified interaction pattern: tiger-following (light red, forward diagonal lines), leopard-following (light blue, backward diagonal lines), bidirectional following (light purple, cross-hatching), avoidance (light green, dotted), and no overlap (light gray, circular hatching). Empty cells indicate partitions without shared data for the corresponding pair. Right-margin labels mark the pair-level interaction types, and thick horizontal lines separate different types of interaction dynamics

Across all 91 valid pair-partitions from the 17 tiger-leopard pairs, three states dominate the interaction dynamics (Fig. 2). For visualization, we split the following patterns into directional subcategories: *tiger-following* and *leopard-following*. Overall, *avoidance* is the most common outcome (35 partitions, *38.6%*). Directional following is also frequent, with leopard-following and tiger-following each occurring in 22 partitions (24.1%). *Bidirectional* following is rare, accounting for only 1 partition (*1.1%),* while *no overlap* explains the remaining 11 partitions (*12.1%*).

*High-conflict dynamics* are observed in only one pair (*5.9%*), which displays frequent concurrent and delayed interactions, including at least one “bidirectional following”. *Stable coexistence* is observed in six pairs (*35.3%),* with sustained home range overlap and a mixture of leopard-following (*31.5%*), tiger-following (*33.3%*), and avoidance (*35.2%*). The remaining ten pairs (*58.8%)* exhibit *home range shift competition*, with avoidance being the dominant local state (*48.5%*), followed by no overlap (*33.3%*), leopard-following (*12.1%*), and tiger-following (*6.1%*). This pattern shows moderate to low interaction frequencies that culminate in home range shift, often ending with “avoidance” or “no overlap”. Dyad-level sample sizes and event summaries are provided in Appendix Table F8. Extended dyad-level figures for all 17 tiger-leopard pairs are provided in Supplementary File 2.

To further illustrate these aggregate patterns and examine potential ecological contexts of each interaction type, we highlight three representative case studies drawn from four individuals (Table 3). These case studies are used as illustrative examples of pairwise time-geographic patterns rather than definitive evidence of behavioral motivation.

**Table 3:** Summary of GPS tracking data and home range sizes of tigers and leopards used in the case study. Fix Count indicates the number of valid GPS locations recorded in the data

ID	Species	Sex	Fix Count	Start Date	End Date	Home Range (km$^2$)
FT1	Tiger	Female	18,584	2018–08-30	2020–11-16	140.7
MT3	Tiger	Male	8,625	2019–09-12	2020–10-02	201.8
ML2	Leopard	Male	3,470	2019–10-09	2020–03-28	59.5
ML4	Leopard	Male	9,507	2019–10-19	2021–01-06	162.6

### High-conflict dynamics

Only one pair, MT3–ML2 (male tiger MT3; male leopard ML2), shows high-conflict dynamics. MT3 is the subadult male (“son”) reported in Liu et al. [14]. In partitions 1–3 (approximately during its first three months), he remains within his mother’s home range, dispersing starting from partition 4.

**Fig. 3:**
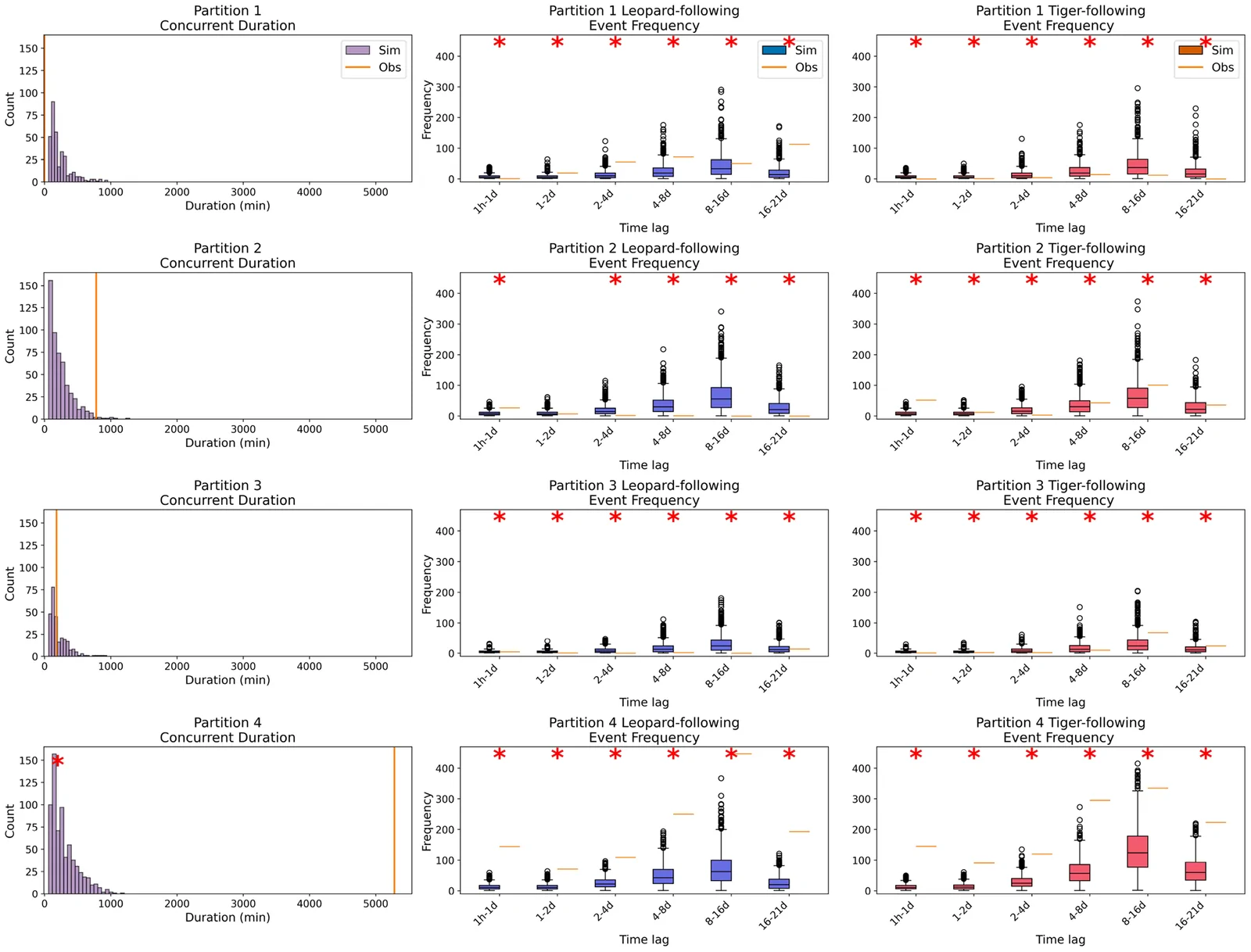
Null-model tests of interaction patterns between MT3 (tiger) and ML2 (leopard) over time. Left column: concurrent interaction durations, with simulated values from 1,000 CRW replicates (purple histograms) compared against the observed total (orange line). Middle column: leopard-following event frequencies, with simulated distributions (blue boxplots) versus observed counts (orange line). Right column: tiger-following event frequencies, with simulated distributions (red boxplots) versus observed counts (orange line). Red stars mark significant deviations from the null (*p < 0.01*)

Figure 3 shows the null-model test results across partitions. In the left panel, purple histograms represent simulated concurrent durations under 1,000 CRW replicates, with orange vertical line marking the observed totals. Partition 4 shows observed concurrent duration significantly exceeding the null expectation (*p < 0.01*), whereas partitions 1 and 3 fall within null expectations. This indicates that the strongest concurrent interaction signal is concentrated in the final observed partition.

The center and right columns in Fig. 3 illustrate directional following dynamics. Boxplots display simulated event frequencies (blue = leopard-following, red = tiger-following) compared against observed values (orange lines). The red stars on the top of the boxplots denote significant deviations from null expectations. Across most time-lag bins and partitions, the observed event frequencies depart significantly from the CRW null model, revealing non-random directional reuse of previously visited areas. In partition 1, the leopard consistently more frequently follows the tiger, while tiger-following is absent. The results suggest frequent leopard-following at lags up to 8 days, possibly to scavenge kills, without reciprocal response. In partitions 2 and 3, however, this pattern reverses: tiger-following dominates at longer lags especially around 8–16 d and 16–21 d, suggesting that the tiger increasingly reused areas previously visited by the leopard. By partition 4, both directions are significantly elevated across nearly all lag bins, extending from 1 h to 21 d, reflecting a bidirectional following dynamic, with tiger-following slightly more common. Because ML2’s data terminate immediately after partition 4, we cannot determine whether the subsequent absence reflects collar failure, collar removal, mortality, or movement beyond the monitored period. We therefore interpret this final high-conflict partition cautiously.

**Fig. 4:**
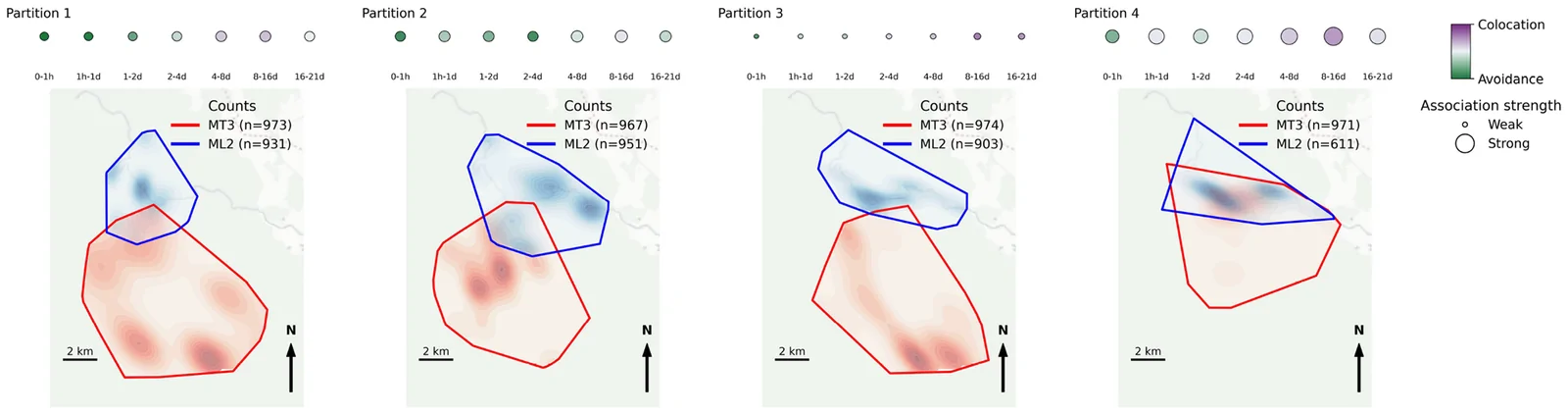
Combined visualization of interaction dynamics between MT3 (red outlines and light red kernel-density shading) and ML2 (blue outlines and light blue kernel-density shading) across sequential partitions. For each partition, the lower panel shows species-specific home range boundaries and kernel-density utilization distributions, illustrating spatial overlap and intensity of core use areas. The upper circle strip summarizes interaction indices across time lags, where circle color represents the relative interaction intensity *R* (green = avoidance, purple = colocation/encounter) and size is proportional to the composite magnitude of interaction *M* (larger = stronger, smaller = weaker). Increasing overlap and stronger purple circles in partition 4 highlight intensified encounters

The combined interaction-index and home range visualization (Fig. 4) captures the escalation of interaction between MT3 and ML2. Over successive partitions, interaction intensity (*R*) strengthens from weak, short-lag associations to strong, sustained overlap across multi-day lags, while the magnitude index (*M*) peaks in partition 4. Spatially, MT3’s range expands northward into ML2’s territory. This pattern indicates a shift from limited early overlap toward stronger delayed colocation in the final observed partition, although the behavioral mechanism underlying this shift cannot be determined from the pairwise patterns alone.

**Fig. 5:**
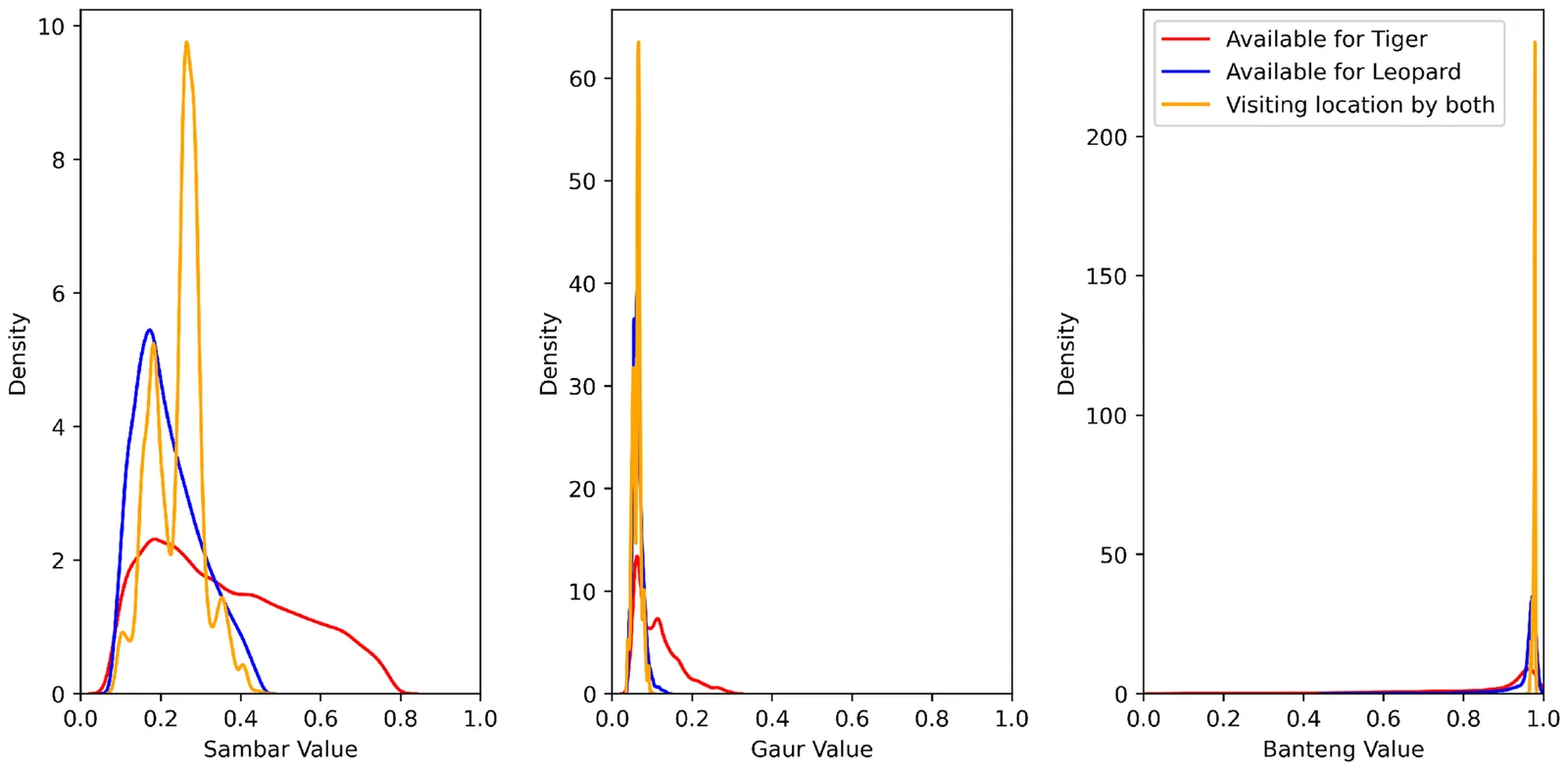
Kernel density distributions of occupancy values are shown for Sambar, Gaur, and Banteng. Red curves = prey distribution within the tiger’s range; blue = prey distribution within the leopard’s range; orange = prey values at locations visited by both species during delayed interactions

Finally, prey distribution analysis (Fig. 5) provides ecological context for these dynamics. We only consider occupancy values above 0.6 as ecologically supportive [41]. Sambar occupancy exceeds 0.6 only in the tiger’s range, Gaur never reaches this threshold in any range, while Banteng habitat remains suitable across both individual and shared ranges. This suggests that MT3 has access to mapped Sambar-supportive areas, whereas ML2’s mapped large-bodied prey context was more strongly associated with Banteng. Because smaller leopard prey were not included in the prey layers, this pattern is interpreted as resource-context evidence rather than a complete prey-availability assessment.

### Stable coexistence

Stable coexistence is the dominant tiger-leopard interaction type among pairs with longer overlapping timeline, observed in six dyads: FT1–ML1, FT1–ML4, FT3–ML1, MT1–ML4, MT2–ML4, MT3–ML4. Here we highlight FT1–ML4 (female tiger FT1; male leopard ML4) as a representative case. FT1 is the mother tiger reported in Liu et al. [14]. Her offspring disperse after partition 3, yet her core movements remain remarkably consistent thereafter. For brevity, we present only the first four partitions here, later partitions are provided in the Supplementary File 2.

**Fig. 6:**
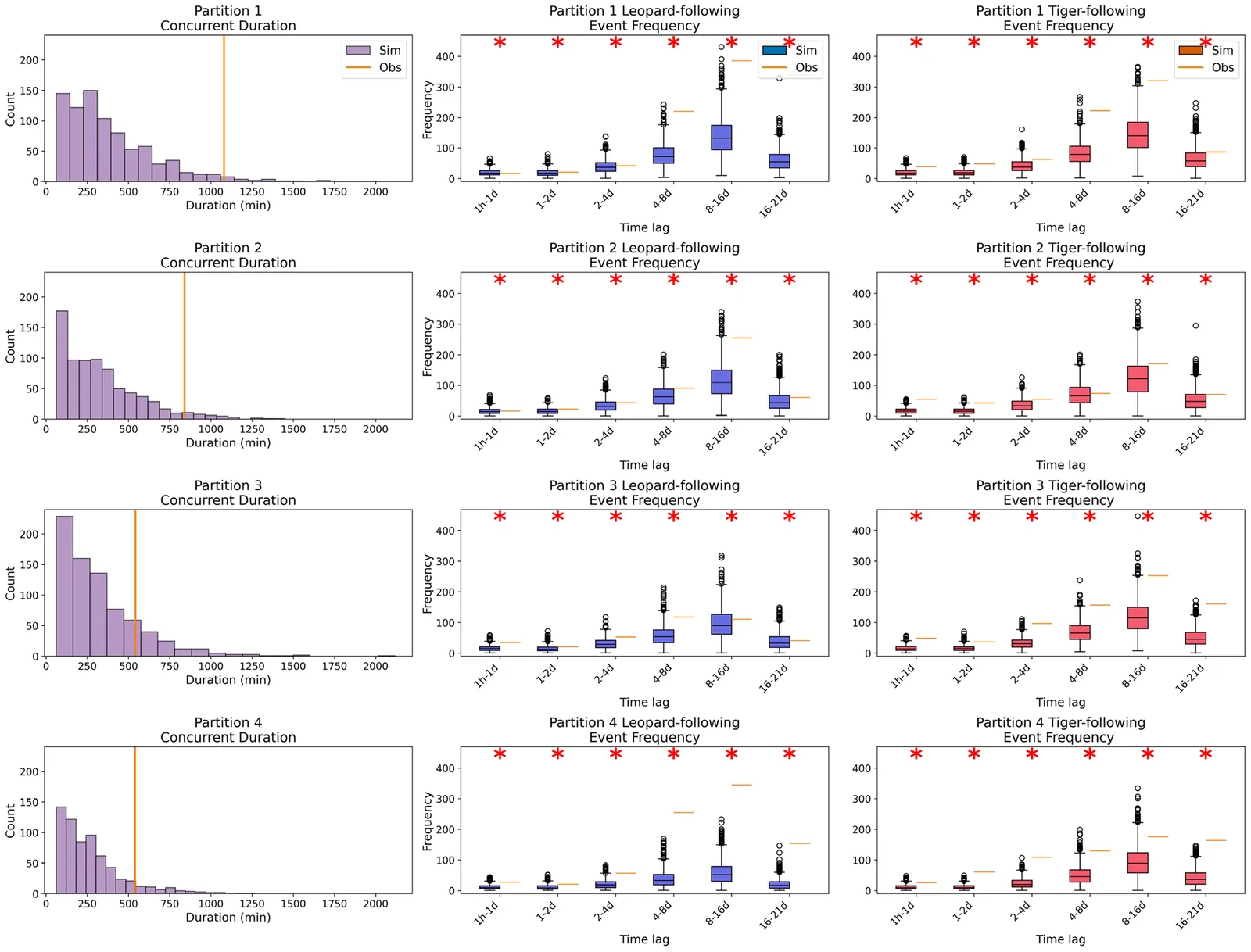
Null model results for FT1 and ML4, following the same layout and color conventions as Fig. 3. Stars denote significant *p < 0.01* differences between observed and simulated concurrent durations or directional following frequencies

Figure 6 shows the null-model comparison of concurrent duration and directional following frequencies. Observed concurrent durations do not show significant deviations from the simulated distributions across the first four partitions. In contrast, directional following frequencies differ from the null expectation across many lag bins in both directions. Although tiger- and leopard-following alternate in direction, the strongest association patterns occur at multi-day lags, especially around 4–16 d, while immediate concurrent interaction remains limited. This pattern is consistent with temporal separation within a persistently shared landscape.

**Fig. 7:**
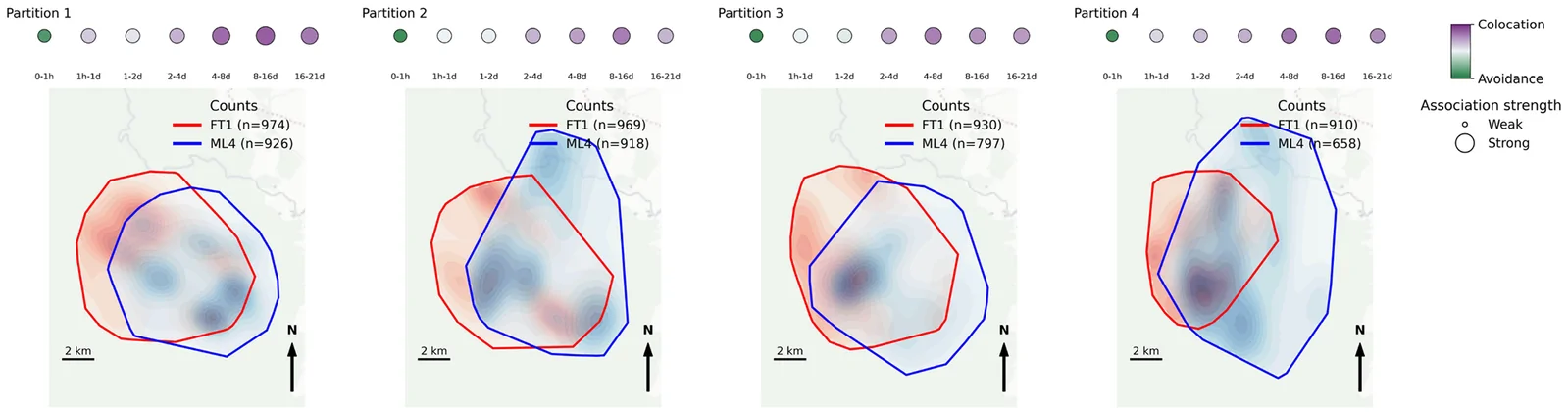
Combined interaction-index and home-range visualization for FT1 (red) and ML4 (blue) across partitions 1–4, using the same layout as Fig. 4. Persistent purple circles at multi-day lags and stable spatial overlap indicate long-term coexistence maintained through temporal partitioning

The combined interaction-index and home range visualization (Fig. 7) shows persistent spatial overlap accompanied by temporal separation. Across four partitions, purple circles are most evident at multi-day lags, especially around 4–16 d, whereas shorter lags show weaker colocation or avoidance tendencies. Unlike the high-conflict case, this pattern remains spatially stable over time, suggesting that FT1 and ML4 take turns to visit the same location by one to two weeks, suggesting repeated delayed use of shared areas without frequent immediate overlap. Both species concentrate in shared cores across partitions, consistent with their temporal partitioning and sustained delayed interactions.

**Fig. 8:**
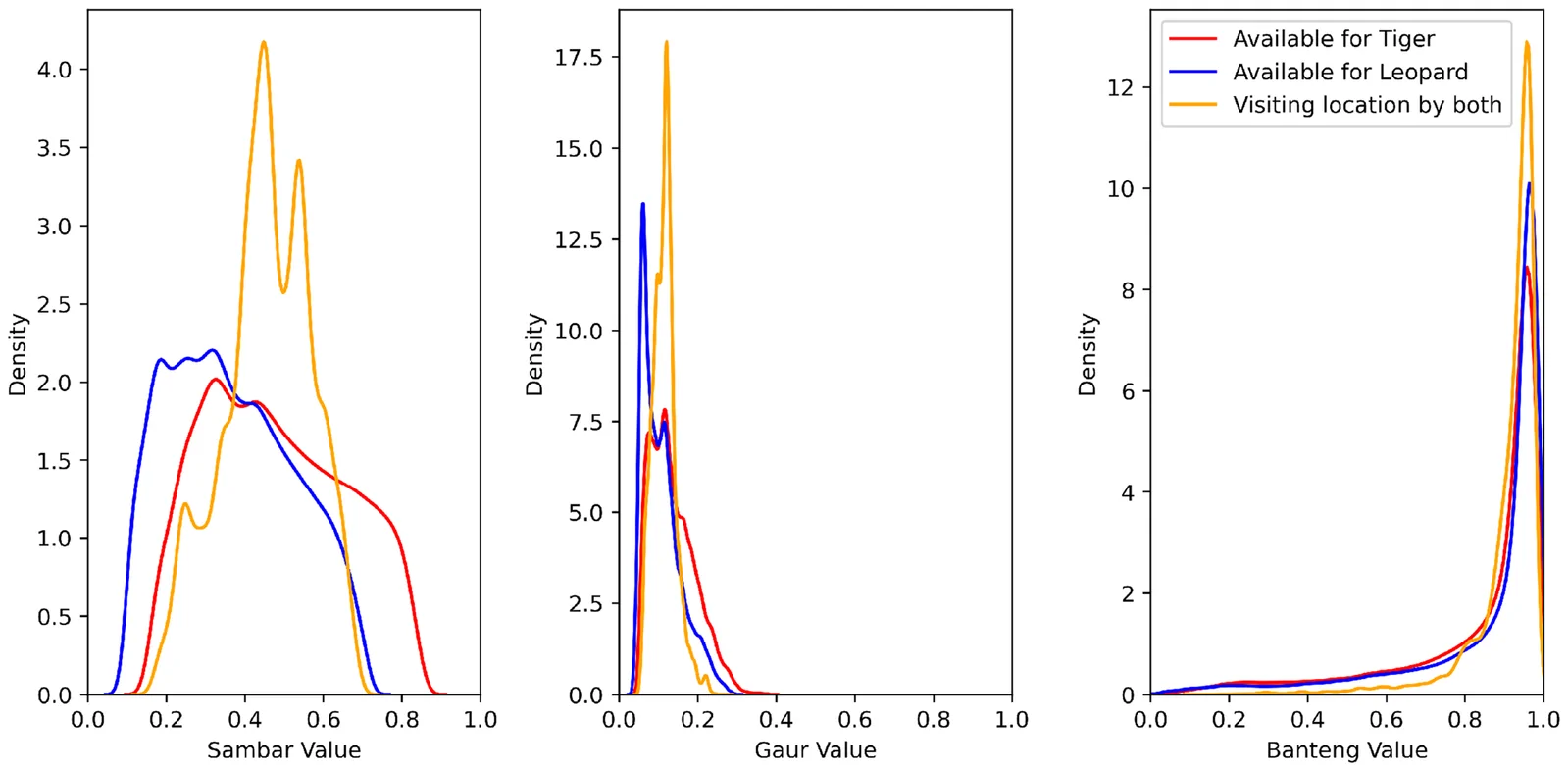
Prey occupancy distributions for FT1 and ML4, using the same configuration as Fig. 5. Only Sambar and Banteng exceed the 0.6 suitability threshold, providing mapped prey context for repeated delayed colocation in shared patches

Finally, Fig. 8 compares prey-occupancy distributions. Both home ranges exceed the 0.6 suitability threshold for Sambar and Banteng, whereas Gaur remains marginal. This mapped large-bodied prey context is consistent with repeated delayed colocation at similar lags, as both predators may use the same high-value foraging areas asynchronously.

### Home range shift competition

Home range shift competition is the dominant type among pairs with short overlapping timelines, observed in ten dyads: FT1-FL, FT1–ML2, FT1–ML3, FT2–ML1, FT3-FL, MT2-FL, MT2–ML1, MT2–ML3, MT3–ML1, MT3–ML3. We use FT1–ML2 (female tiger FT1; male leopard ML2) as a representative case. FT1 is the same mother tiger described in Section “Stable coexistence”, and ML2 is the same male leopard mentioned in Section “High-conflict dynamics”.

**Fig. 9:**
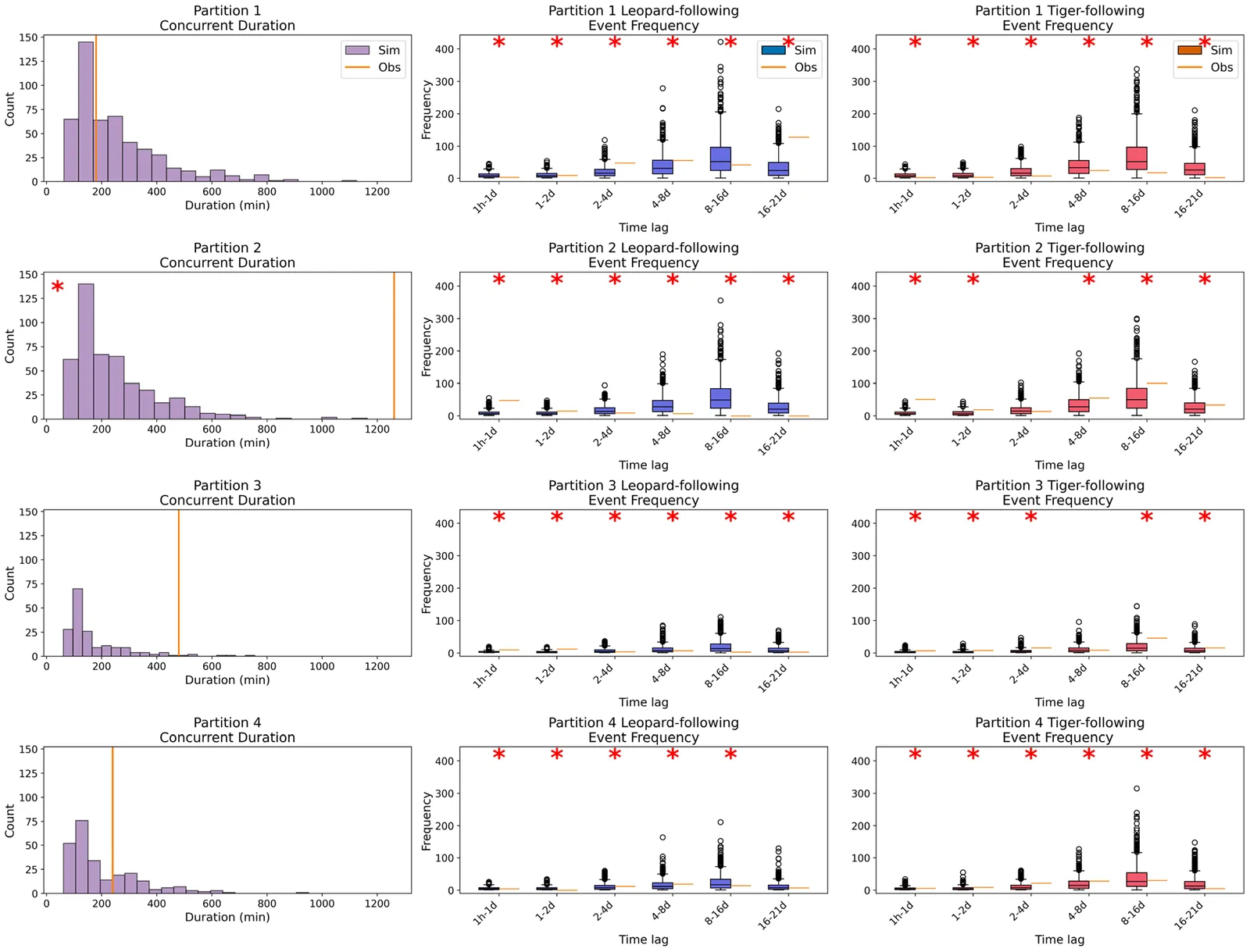
Null model results for FT1 and ML2, following the same layout and color conventions as Fig. 3. Stars denote significant *p < 0.01* differences between observed and simulated concurrent durations or directional following frequencies

Figure 9 shows that, in partition 1, although concurrent durations fall within null expectations, leopard following frequency is significantly higher at 2–4 d and peaks at 16–21 d. In partition 2, observed total concurrent duration exceeds the simulated distributions, indicating elevated concurrent interaction between tiger and leopard, accompanied by significantly higher tiger-following frequency at 8–16 d. In partition 3 and 4, concurrent durations fall within null expectations. Directional following tests still show deviations from the null in several lag bins, but the observed frequencies and interaction-index patterns indicate weaker associations as spatial overlap declines.

**Fig. 10:**
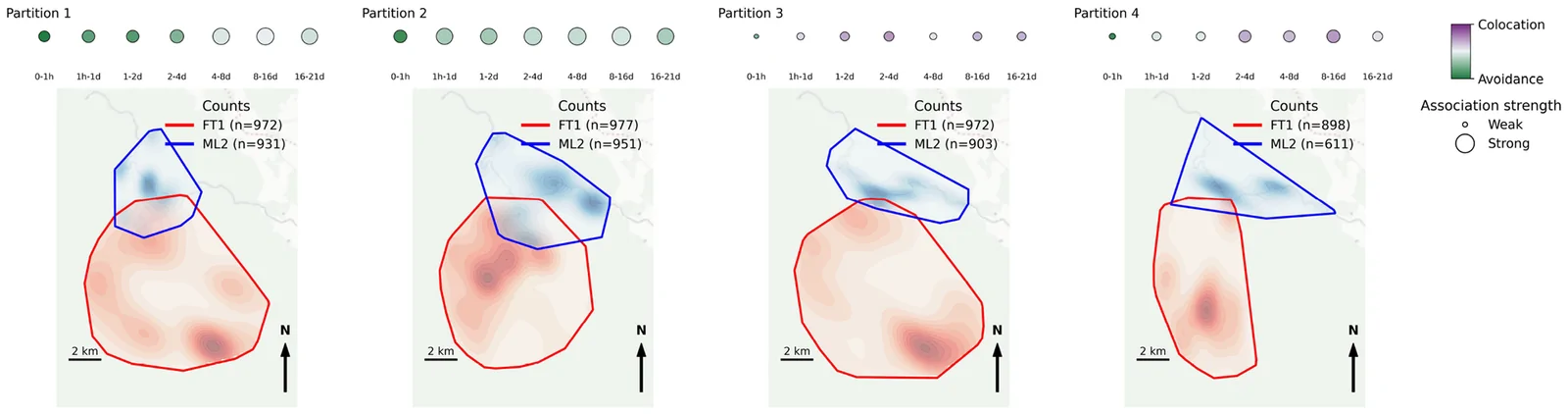
Combined interaction-index and home-range visualization for FT1 (red) and ML2 (blue) across sequential partitions, using the same layout as Fig. 4. Shrinking purple circles and reduced overlap illustrate declining interaction intensity and an apparent home-range shift by ML2

The combined interaction-index and spatial analysis (Fig. 10) illustrates how weakening interactions coincide with reduced spatial overlap. In partitions 1–2, larger purple circles at longer lags (4–16 d) and overlapping home ranges indicate stronger delayed colocation, whereas smaller green circles at shorter lags (*≤* 4 h) reflect reduced short-lag association rather than immediate colocation. By partitions 3–4, both circles and overlap area shrink markedly as ML2 shifts its activity center away from FT1’s stable core. This concurrent decline in interaction magnitude and spatial overlap is consistent with a transition from shared space use to reduced overlap, rather than definitive evidence of competitive segregation.

**Fig. 11:**
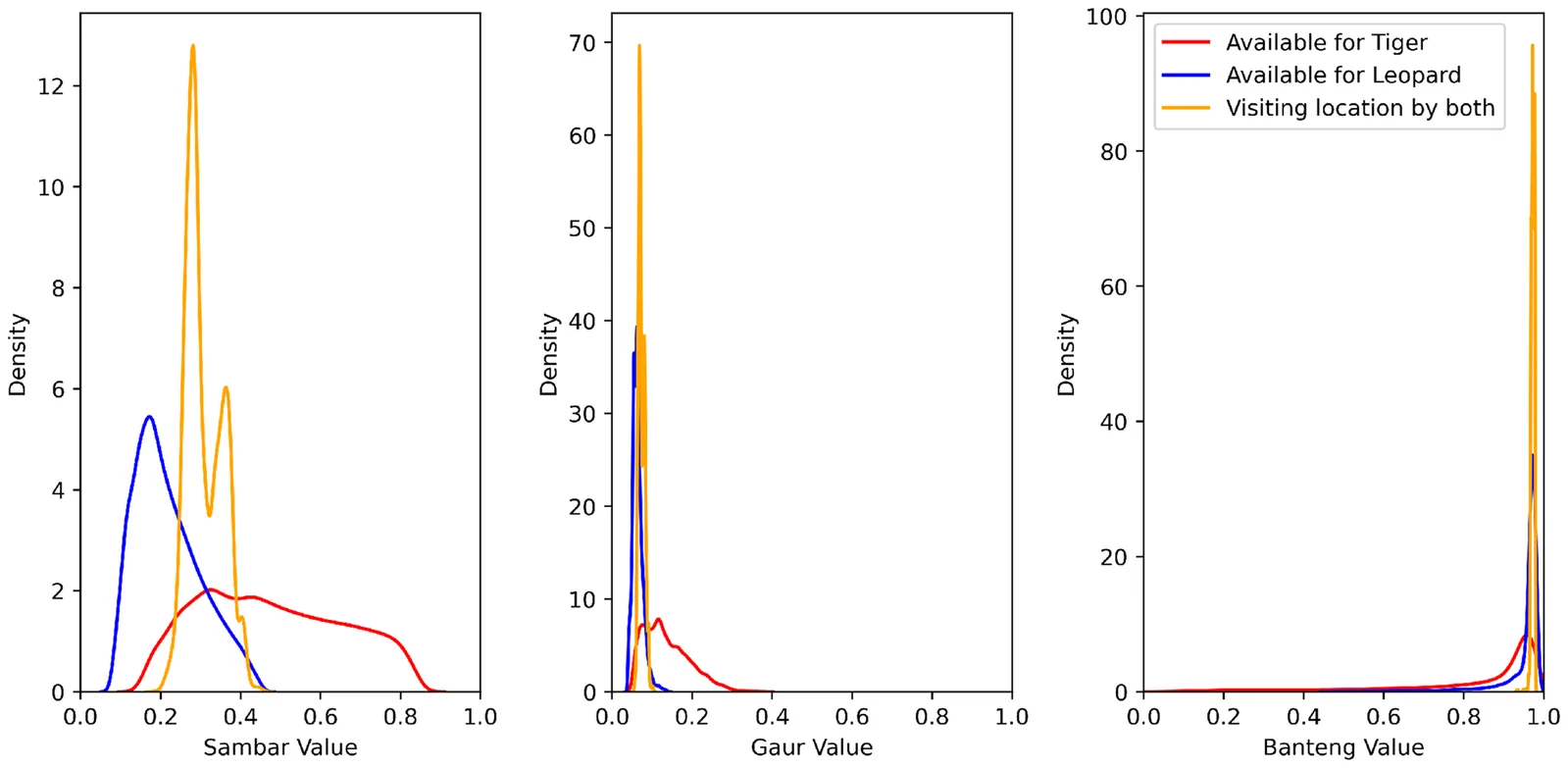
Prey occupancy distributions within FT1 and ML2 ranges, using the same configuration as Fig. 5. High mapped Banteng occupancy provides ecological context for shared but temporally separated use, while Sambar occupancy above the threshold occurs mainly in the tiger range

Finally, mapped large-bodied prey context patterns (Fig. 11) provide ecological context for this interpretation. Banteng occupancy is high in both home ranges, while Sambar exceeds the threshold only in the tiger’s range. Locations jointly visited during delayed interactions occur in high-Banteng areas, suggesting that mapped shared prey context may contribute to week-scale delayed use. However, because smaller leopard prey and neighboring individuals are not represented in this figure, alternative explanations for the apparent shift are considered in the Discussion.

## Discussion

Across 17 tiger-leopard pairs, we identify two broader recurring interaction patterns: stable coexistence and home range shift competition, together with one rare high-conflict case. These patterns describe structured pairwise spatiotemporal associations rather than direct behavioral motivation. They suggest that tiger-leopard coexistence is shaped jointly by movement opportunity, mapped large-bodied prey context, life-history stage, and the broader territorial setting in which each dyad is embedded.

### Ecological drivers of global interaction dynamics

Life-history stage and mapped prey context may contribute to high-intensity interactions. The high-conflict case (MT3–ML2) occurs as a subadult male tiger expanded from its natal range into the activity area of an adult male leopard, coinciding with frequent encounters and bidirectional following (Figs. 3, 4). Such a pattern is consistent with territorial instability of dispersing subadults and interference interactions recorded in Nepal population and the same Thai populations [5, 42].

Two non-exclusive mechanisms may explain this escalation. First, the available prey layers indicate that Banteng is the only mapped large-bodied prey species consistently above the occupancy threshold for both individuals (Fig. 5). This may concentrate both predators within the same broad resource context, increasing opportunities for repeated interaction [1, 6, 7, 48]. However, because our prey layers do not include smaller prey important to leopards, this should be interpreted as mapped large-prey context rather than complete prey limitation [4, 49]. Second, the tiger’s subadult dispersal stage may have increased territorial uncertainty and exploratory movement, while the resident leopard may also have responded actively to the tiger’s arrival. We therefore interpret MT3–ML2 as a rare high-intensity case rather than evidence that subadult tigers generally over-respond to resident leopards.

In contrast, the stable coexistence case (FT1–ML4) illustrates how shared landscapes can be used asynchronously when mapped large-bodied prey resources are broadly available (Figs. 6, 7). Here, both Sambar and Banteng exceed the occupancy threshold across shared ranges (Fig. 8), suggesting that neither species needs to concentrate exclusively a single mapped large-prey resource. Instead of frequent immediate overlap, the pair repeatedly uses shared areas with delays of approximately 8–16 days. This pattern is consistent with the temporal and spatial niche partitioning documented between tigers, leopards, and other large carnivores in prey-rich systems [1, 6, 7].

A third pattern, represented by FT1–ML2, captures the transition from shared space use to reduced overlap. Early partitions show significant delayed interactions, but overlap declines rapidly as the leopard shifts its activity center away from the tiger’s stable core (Fig. 10). This pattern may reflect competitive displacement, but alternative explanations are also plausible, including cub-rearing behavior by the female tiger, attraction of the leopard to tiger kill sites, responses to neighboring individuals, or broader territorial dynamics not represented by this pair. Similar uncertainty is common in dominance-asymmetric carnivore systems [1, 7, 50, 51], where subordinate species may avoid dominant competitors, track resources, or maintain coexistence through spatial and temporal partitioning.

### Temporal structure of following and avoidance behaviors

Across stable-coexistence pairs, both leopard-following and tiger-following counts at 4–21 d lags are significantly and consistently above null expectations. These long-lag revisits suggest structured reuse of areas recently used by the other species, potentially indicating an awareness of the presence of each other. Such patterns align with mechanisms like territory patrolling [42] and scent-mark detection [48, 52], where persistent long-lag following can help each tiger/leopard monitor neighbor activity without risking direct confrontation. In addition, both species revisit the same prey-rich areas on multi-day cycles consistent with memory of resource locations (prey occupancy).

In contrast, interactions at short lags (0–24 h) are typically skewed towards avoidance. The rarity of same-day encounters suggests that direct conflict is actively avoided, again consistent with scent-mediated spatial segregation along territorial boundaries [48, 52]. Together, these results highlight how indirect communication and memory of shared resources underpin coexistence strategies in this multi-carnivore system.

### Implications, limitations, and future work

Our findings underscore that coexistence between large carnivores is not a single outcome but a spectrum of strategies modulated by prey landscapes and life-history stage. From a conservation perspective, maintaining a diverse prey base and connected habitats may reduce direct conflict and buffer against displacement, especially in landscapes where apex predators coexist [1, 7]. This interpretation is consistent with broader evidence that dominance-asymmetric carnivore systems do not follow a single coexistence mechanism. In some systems, dominant carnivores can suppress subordinate competitors, as documented for wolves and coyotes [53]. In other systems, subordinate carnivores can persist with limited detectable population-level displacement, as shown for lions and leopards in parts of Africa [51]. Tiger-leopard coexistence may similarly involve a mixture of risk avoidance, temporal partitioning, resource tracking, and individual-specific territorial responses rather than one fixed mechanism.

Methodologically, the time-geographic framework combined with null-model testing and interaction indices offers a structured way to disentangle intentional interactions from random overlap. Sensitivity analysis shows that the interaction detection and null-model comparison are robust to temporal partition length, with the 1,000 h window serving mainly as a temporal summarization unit (Figure B2). However, several limitations shape how these pairwise patterns should be interpreted. First, although the telemetry dataset is detailed for wide-ranging carnivores, it includes a limited number of individuals and 17 tiger-leopard dyads. The high-conflict pattern is represented by one dyad, so we interpret it as a rare high-intensity case rather than a broadly generalizable archetype. Each pair was also embedded within a larger community of neighboring tigers, leopards, prey, and territorial boundaries, meaning that the detected patterns may partly reflect broader multi-individual context rather than only the pairs.

Second, the intentionality analysis involves many pair-partition-lag comparisons. We addressed this by adopting a conservative threshold of *α=0.01* for assigning local interaction states (Appendix D). However, individual lag-specific tests should still be interpreted cautiously, especially when sample sizes are small or when multiple lag bins show mixed evidence [46, 47]. The knowledge-guided bidirectional-following rule used in the pair-level classification should also be viewed as a refinement that improves consistency with expert-informed reference labels, rather than independent evidence of perfect classification performance.

Third, the CRW null model is movement-based rather than resource-conditioned. Although it preserves movement structure and feasible domains, it does not explicitly incorporate habitat selection or prey occupancy. If both species independently concentrate movement around productive prey patches, the movement-only null model may underestimate expected overlap in those areas and make some delayed colocation patterns appear more intentional than they would under a resource-aware null. Future work could address this limitation by generating habitat-informed null trajectories using step-selection or integrated step-selection simulations before applying the same interaction analysis [38, 54].

Finally, the prey layers used here represent mapped large-bodied ungulate prey context rather than complete prey availability. This limitation is especially important for leopards, which can use a broader prey spectrum and often rely on smaller or younger prey than adult Sambar, Gaur, or Banteng [4, 49, 55]. Therefore, the prey layers are best interpreted as broad ecological context rather than direct evidence of prey-driven interaction mechanisms. The ecological interpretation is also specific to the HKK protected-area system, where tiger-leopard coexistence occurs within a relatively intact prey and carnivore community. In addition, GPS sampling intervals of 15–60 min may miss very fine-scale encounters, short chases, or rapid avoidance responses. Our analysis therefore emphasizes multi-hour to multi-day interaction structure rather than immediate behavioral encounters, and future studies with higher-frequency GPS data or biologging sensors could better resolve these fine-scale responses [56, 57].

Future work could explore alternative randomization schemes, habitat-informed null models, and additional environmental covariates. Our approach demonstrates the value of integrating spatial, temporal, and ecological dimensions to uncover interaction pattern, while also highlighting that statistical deviations from a null model should be interpreted within species-specific ecological context.

## Conclusion

By using a time-geographic framework and pairing it with correlated random walk null models, this research reveals that tiger–leopard interactions are not uniform but vary across pairwise contexts. We identify two broader recurring patterns, stable coexistence and home range shift competition, together with one rare high-conflict case. High-conflict case reflects the combined influence of subadult dispersal, territorial uncertainty, and mapped large-bodied prey context. Stable coexistence, the dominant pattern, occurs where both species maintain shared space use while delaying visits by several days, suggesting temporal partitioning rather than strict spatial segregation. Home range shift competition reflects a transition from shared space use to reduced overlap, with delayed colocation weakening as spatial overlap declines. Overall, these patterns suggest that coexistence depends on both ecological context and individual life history. For conservation, maintaining prey diversity and habitat connectivity will be critical to reduce conflict and promote long-term coexistence between these two apex carnivores.

## Electronic supplementary material

Below is the link to the electronic supplementary material.

Supplementary Material 1

Supplementary Material 2

## Data Availability

The GPS tracking data used in this study were collected by the coauthors and are not publicly available due to the sensitive nature of endangered species locations and associated poaching risk. Access to these data is restricted by the data owners and may be considered upon reasonable request, subject to appropriate permissions and a data-use agreement. Analysis code and non-sensitive derived data products are made publicly available at GitHub https://github.com/move-ucsb/tlg-avoid.
