# Supplementary material for: Avoidance dynamics in tiger-leopard coexistence: a time-geographic approach
Source: Mov Ecol. 2026 Jul 20;14:72. doi: 10.1186/s40462-026-00681-7 (PMC13673898; doi:10.1186/s40462-026-00681-7)
Supplement: Supplementary file 1 [file 40462_2026_681_MOESM1_ESM.pdf]

Appendix A Full dataset description and study area figure

We utilized GPS tracking data collected from six Indochinese tigers (three females and three males) and five leopards (one female and four males), with sampling intervals ranging from 15 minutes to one hour (h) (Figure A1). The tracking duration varies from four months to 28 months for each individual. Female tigers contributing between 6,151 and 18,584 GPS points, and male tigers contributing between 7,164 and 8,625 points. Similarly, the female leopard contributed 8,034 points, while the four male leopards contributed between 2,257 and 10,717 points.

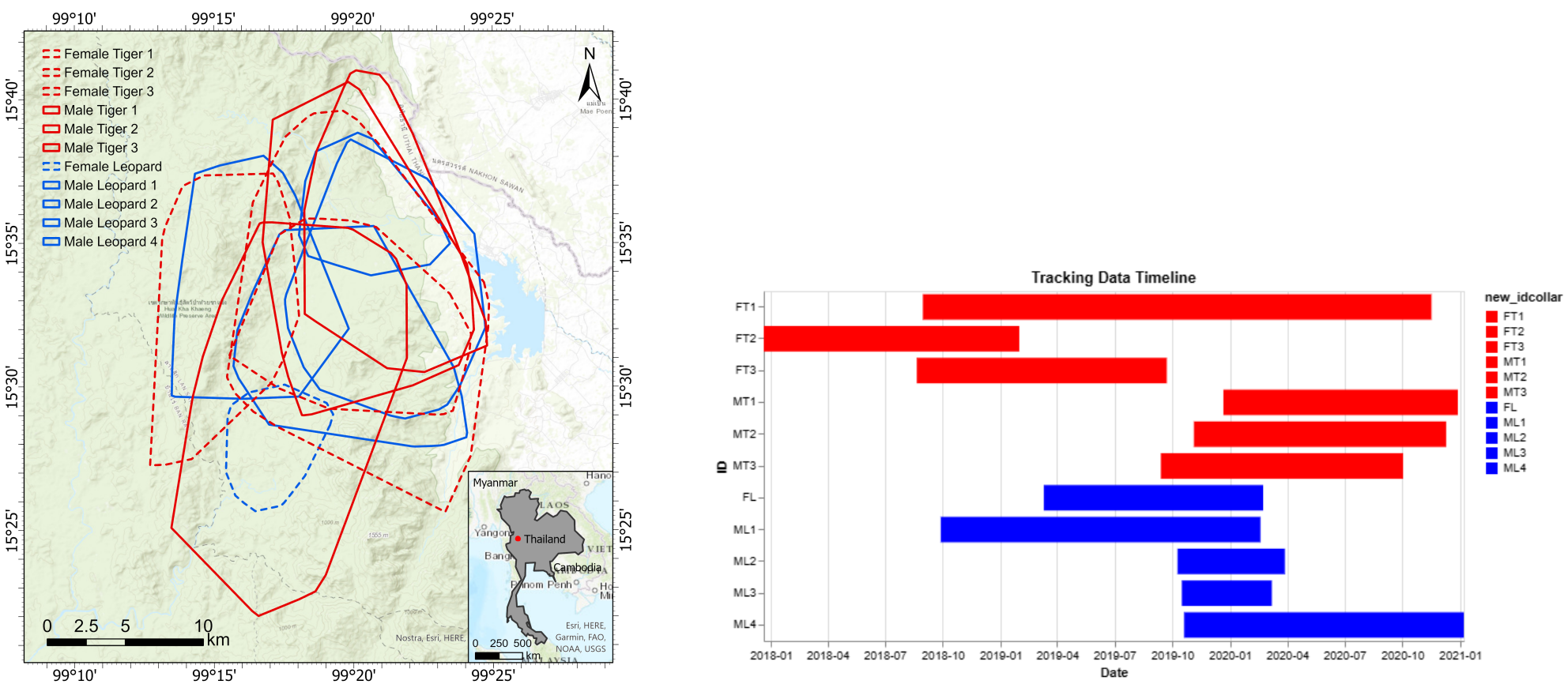

(a) Study area (b) Timeline

Fig. A1: (a) Study area and (b) timeline of six tigers (three females in red dashed line, labelled as FT; and three male in red solid line, labelled as MT) and five leopards (one female in blue dashed line, labelled as FL; and four male in blue solid line, labelled as ML) collected between 2018 and 2021.

The home range sizes of the tigers exhibit significant variability, reflecting shifts in resource selection and behavioral interactions (Table 3, Figure A1b). Male tigers generally have home ranges approximately three times larger than female tigers, and these spatial extents fluctuate monthly, likely in response to environmental factors

ID	Species	Sex	Fix Count	Start Date	End Date	Home Range (km^2)
FT1	Tiger	Female	18,584	2018-08-30	2020-11-16	140.7
FT2	Tiger	Female	9,504	2017-12-20	2019-01-30	133.3
FT3	Tiger	Female	6,151	2018-08-20	2019-09-22	290.6
MT1	Tiger	Male	7,164	2019-12-21	2020-12-28	148.7
MT2	Tiger	Male	7,786	2019-11-04	2020-12-09	263.0
MT3	Tiger	Male	8,625	2019-09-12	2020-10-02	201.8
FL	Leopard	Female	8,034	2019-03-10	2020-02-22	39.8
ML1	Leopard	Male	10,717	2018-09-27	2020-02-18	152.9
ML2	Leopard	Male	3,470	2019-10-09	2020-03-28	59.5
ML3	Leopard	Male	2,257	2019-10-16	2020-03-07	131.5
ML4	Leopard	Male	9,507	2019-10-19	2021-01-06	162.6

Table A1: Summary of GPS tracking data and home range sizes of six tigers and five leopards.

such as prey density (Smith et al. 1987; Simcharoen et al. 2022) and the presence of other large carnivores (Liu et al. 2024b).

Appendix B Sensitivity to temporal partition length

To evaluate whether the ORTEGA-based interaction analysis and null-model comparison are robust to temporal partition length, we repeat the interaction analysis for the three focal pairs using 500 h and 2,000 h windows, in addition to the 1,000 h window used in the main analysis. For each pair, comparisons are restricted to the common temporal coverage shared across the three window lengths. We summarize observed concurrent interaction duration and the median concurrent duration from 500 null-model simulations.

Observed concurrent interaction duration is identical across the three partition lengths for each pair, and the null-model median duration is also consistent (Figure B2). These results indicate that the main interaction inference is robust to the computational partitioning scheme. We retain the 1,000 h partition as a practical choice because it balances computational efficiency, temporal organization, and ecological relevance. Tiger scent marks can persist for up to approximately three weeks (Smith

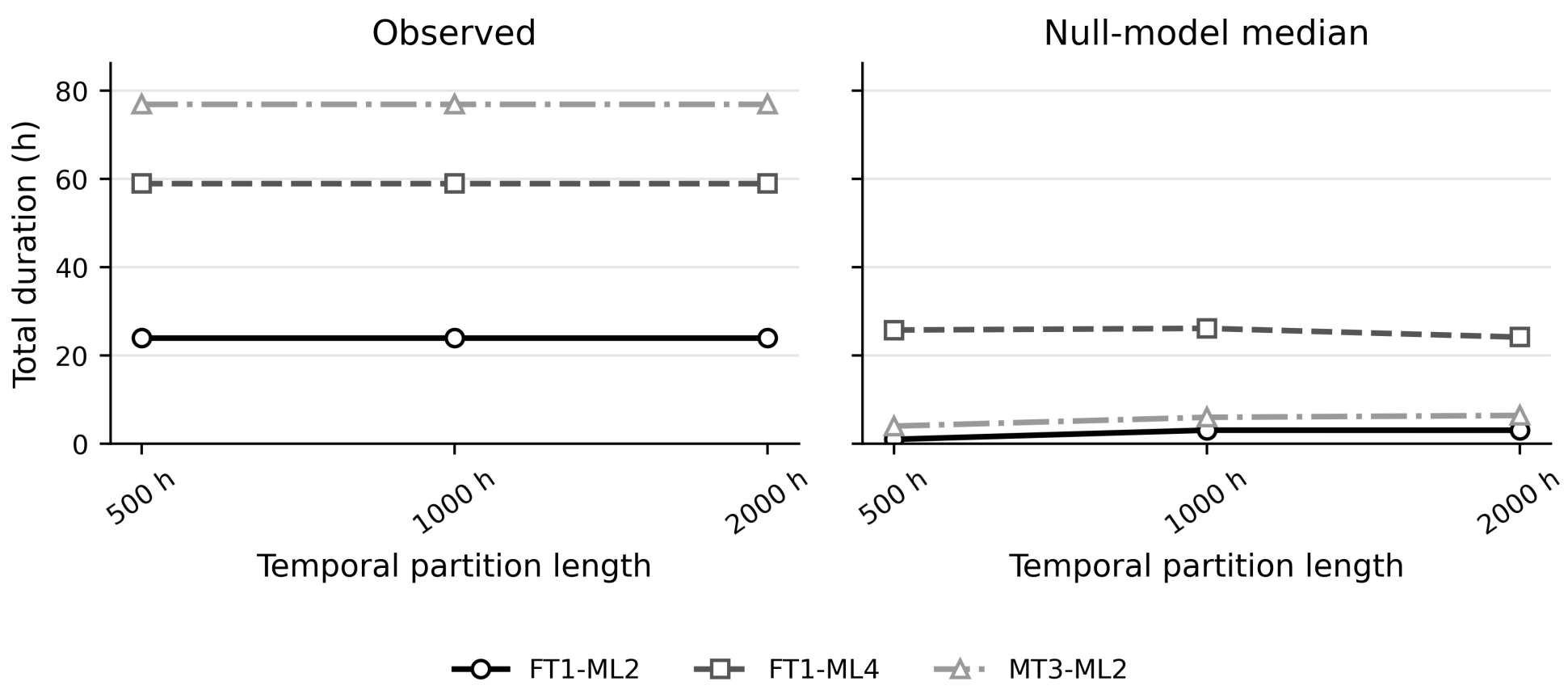

Fig. B2: Sensitivity of concurrent interaction duration to temporal partition length. Observed concurrent interaction duration and null-model median concurrent interaction duration are recalculated using 500 h, 1,000 h, and 2,000 h temporal partitions after restricting each tiger-leopard pair to common temporal coverage.

et al. 1987). Therefore, the 1,000 h window is long enough to include the full delayed-interaction lag range considered in this study, whereas the 500 h window is slightly shorter than 21 days. Temporal partition length determines the resolution at which interaction patterns are summarized and interpreted, but it does not determine the underlying ORTEGA interaction detection or null-model comparison. Shorter partitions can produce more fragmented local summaries, whereas longer partitions can aggregate adjacent behavioral phases.

Appendix C Quadrant diagram for R and M

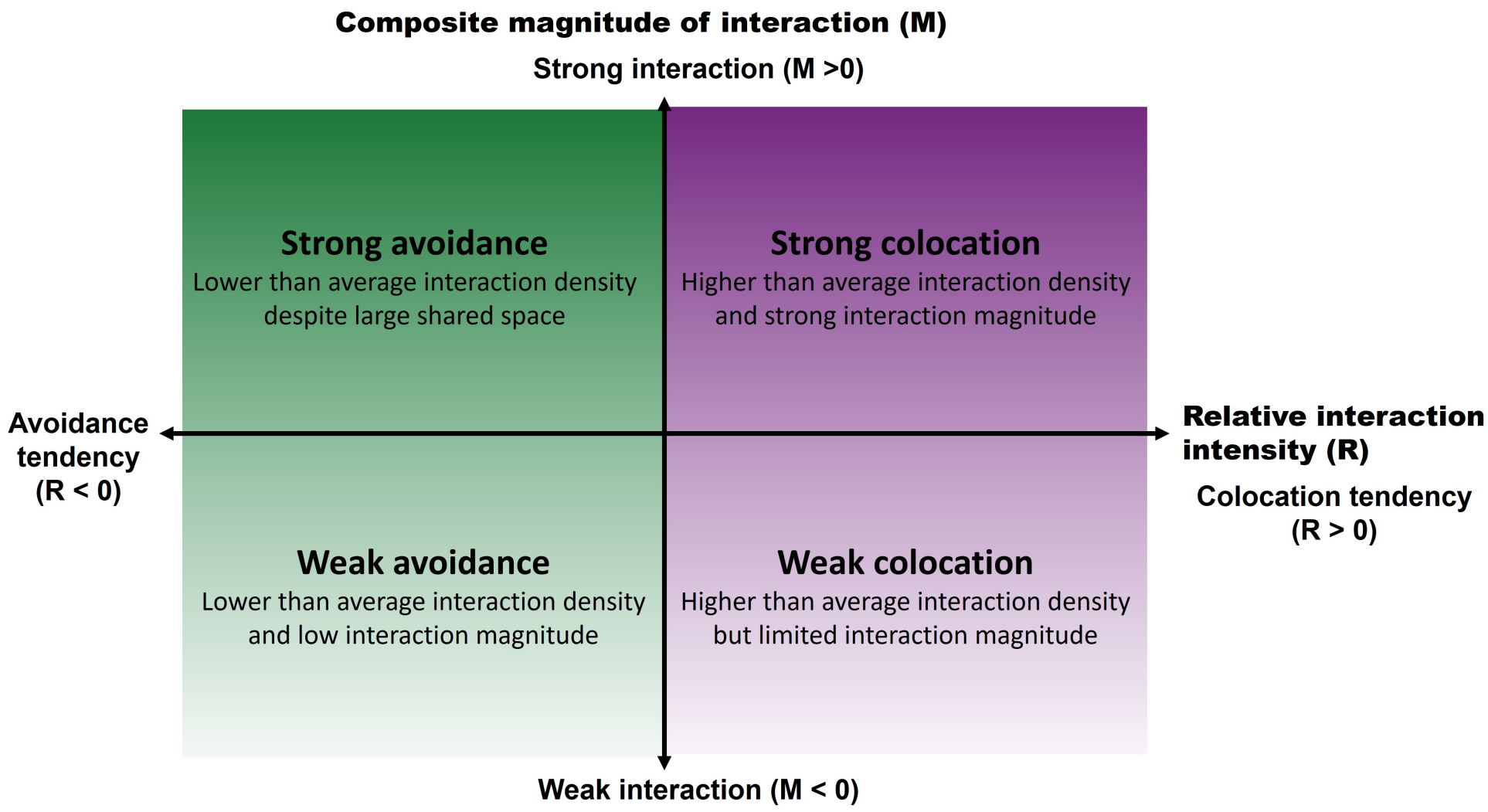

Fig. C3: Schematic interpretation of the two-dimensional interaction space defined by relative interaction intensity (R) and composite magnitude of interaction (M). Positive R values indicate a colocation tendency, whereas negative R values indicate an avoidance tendency. Positive M values indicate stronger interaction magnitude, whereas negative M values indicate weaker interaction magnitude. Together, these indices classify temporal partitions into four quadrants: strong avoidance, weak avoidance, strong colocation, and weak colocation. The composite magnitude index M is derived from dyad-specific standardized values of the proportion of intersected PPAs (P) and overlapped home-range area (A) across valid temporal partitions.

Appendix D Multiple-testing robustness

This appendix compares the number of significant intentionality tests retained under the original $\alpha = 0.05$ threshold, the revised $\alpha = 0.01$ threshold, and Benjamini-Hochberg false-discovery-rate correction (Benjamini and Hochberg 1995; Pike 2011). The revised main analysis uses $\alpha = 0.01$ for assigning local interaction states.

Table D2: Sensitivity of intentionality-test summaries to conservative significance thresholds and Benjamini-Hochberg false-discovery-rate correction.

Criterion	Significant tests	Total tests	Proportion significant
Uncorrected $p \leq 0.05$	93	637	0.146
Uncorrected $p \leq 0.01$	48	637	0.075
Global BH-FDR $q \leq 0.05$	32	637	0.050
Within-lag BH-FDR $q \leq 0.05$	31	637	0.049

Appendix E Pair-level classifier outputs

This appendix summarizes the leave-one-dyad-out classifier outputs used to evaluate whether pair-level interaction dynamics can be reproduced from summary features of partition-level states. The classification procedure is described in Section 3.6.

Table E3: Leave-one-dyad-out predictions from the base multinomial logistic regression classifier and the classifier after applying the bidirectional-following refinement. MT3-ML2 was the only dyad whose base prediction differed from the expert-informed reference label.

Pair	Base classifier label	Label with bidirectional-following refinement
MT3-ML2	Stable coexistence	High-conflict dynamics
FT1-FL	Home range shift competition	Home range shift competition
FT1-ML2	Home range shift competition	Home range shift competition
FT1-ML3	Home range shift competition	Home range shift competition
MT2-ML3	Home range shift competition	Home range shift competition
FT3-FL	Home range shift competition	Home range shift competition
MT3-ML3	Home range shift competition	Home range shift competition
MT3-ML1	Home range shift competition	Home range shift competition
FT2-ML1	Home range shift competition	Home range shift competition
MT2-FL	Home range shift competition	Home range shift competition
MT2-ML1	Home range shift competition	Home range shift competition
FT1-ML1	Stable coexistence	Stable coexistence
FT1-ML4	Stable coexistence	Stable coexistence
FT3-ML1	Stable coexistence	Stable coexistence
MT1-ML4	Stable coexistence	Stable coexistence
MT2-ML4	Stable coexistence	Stable coexistence
MT3-ML4	Stable coexistence	Stable coexistence

Table E4: Leave-one-dyad-out confusion matrices for the base classifier and the classifier with the bidirectional-following refinement. Rows indicate expert-informed reference labels, and columns indicate predicted labels.

**Base classifier**			
Reference label	High-conflict	Home-range shift	Stable coexistence
High-conflict dynamics	0	0	1
Home range shift competition	0	10	0
Stable coexistence	0	0	6

**Classifier with bidirectional-following refinement**			
Reference label	High-conflict	Home-range shift	Stable coexistence
High-conflict dynamics	1	0	0
Home range shift competition	0	10	0
Stable coexistence	0	0	6

Appendix F Temporal diagnostics and aggregate dyad-level summaries

This appendix provides additional diagnostic and aggregate information supporting the Results section. Tables F5-F7 summarize the temporal slicing of the shared tracking period for the three dyads highlighted in the main text. Table F8 provides dyad-level summaries across all 17 tiger-leopard pairs.

F.1 Per-slice diagnostics for case studies in results

The following tables summarize the temporal slicing of the shared tracking period for each dyad, reporting the start and end of each 1,000-h window, the number of GPS fixes per individual, and the median sampling interval.

Table F5: Per-slice diagnostics for MT3 and ML2

Slice	Window start	Window end	MT3	Interval	ML2	Interval
1	2019-10-13 11:00:00	2019-11-24 03:00:00	973	60 min	931	60 min
2	2019-11-24 03:00:00	2020-01-04 19:00:00	967	60 min	951	60 min
3	2020-01-04 19:00:00	2020-02-15 11:00:00	974	60 min	903	60 min
4	2020-02-15 11:00:00	2020-03-28 03:00:00	971	60 min	611	60 min

Table F6: Per-slice diagnostics for FT1 and ML4

Slice	Window start	Window end	FT1	Interval	ML4	Interval
1	2019-11-07 07:00:00	2019-12-18 23:00:00	974	60 min	926	60 min
2	2019-12-18 23:00:00	2020-01-29 15:00:00	969	60 min	918	60 min
3	2020-01-29 15:00:00	2020-03-11 07:00:00	930	60 min	797	60 min
4	2020-03-11 07:00:00	2020-04-21 23:00:00	910	60 min	658	60 min

Table F7: Per-slice diagnostics for FT1 and ML2

Slice	Window start	Window end	FT1	Interval	ML2	Interval
1	2019-10-13 11:00:00	2019-11-24 03:00:00	972	60 min	931	60 min
2	2019-11-24 03:00:00	2020-01-04 19:00:00	977	60 min	951	60 min
3	2020-01-04 19:00:00	2020-02-15 11:00:00	972	60 min	903	60 min
4	2020-02-15 11:00:00	2020-03-28 03:00:00	898	60 min	611	60 min

F.2 Aggregate dyad-level summaries

Table F8 reports dyad-level sample sizes and interaction summaries for all 17 tiger-leopard pairs. These summaries include valid temporal partitions, observed interaction-event counts, partition-state composition, and significant directional-following tests under the revised $\alpha = 0.01$ threshold.

Table F8: Dyad-level sample sizes and observed interaction-event summaries across all 17 tiger-leopard pairs.

Dyad	Pair-level type	Part.	LF	TF	Avoid.	No overlap	Bidir.	Events	Sig. dir. tests
MT3-ML2	High-conflict dynamics	4	1	2	0	0	1	3,206	47/48
FT1-FL	Home range shift competition	8	1	0	0	7	0	29	10/12
FT1-ML2	Home range shift competition	4	1	1	2	0	0	998	45/48
FT1-ML3	Home range shift competition	3	1	0	1	1	0	167	22/24
MT2-ML3	Home range shift competition	2	1	0	1	0	0	181	22/24

Continued on next page

Table F8 continued.

Dyad	Pair-level type	Part.	LF	TF	Avoid.	No overlap	Bidir.	Events	Sig. dir. tests
FT3-FL	Home range shift competition	4	0	1	1	2	0	336	31/36
MT3-ML3	Home range shift competition	3	0	0	2	1	0	25	23/24
MT3-ML1	Home range shift competition	3	0	0	3	0	0	1,160	34/36
FT2-ML1	Home range shift competition	2	0	0	2	0	0	153	19/23
MT2-FL	Home range shift competition	2	0	0	2	0	0	127	24/24
MT2-ML1	Home range shift competition	2	0	0	2	0	0	469	19/24
FT1-ML1	Stable coexistence	12	3	3	6	0	0	7,184	133/143
FT1-ML4	Stable coexistence	9	3	3	3	0	0	8,937	107/108
FT3-ML1	Stable coexistence	8	1	2	5	0	0	1,821	85/96
MT1-ML4	Stable coexistence	8	1	4	3	0	0	2,912	93/93
MT2-ML4	Stable coexistence	9	4	4	1	0	0	2,415	100/108
MT3-ML4	Stable coexistence	8	5	2	1	0	0	7,514	91/96

Note. Part. = valid temporal partitions; LF/TF = leopard-/tiger-following partitions; Avoid. = avoidance partitions; Bidir. = bidirectional-following partitions; Sig. dir. tests = significant directional-following tests / total directional-following tests.
