# Supplementary material for: Avoidance dynamics in tiger-leopard coexistence: a time-geographic approach
Source: Mov Ecol. 2026 Jul 20;14:72. doi: 10.1186/s40462-026-00681-7 (PMC13673898; doi:10.1186/s40462-026-00681-7)
Supplement: Supplementary file 2 [file 40462_2026_681_MOESM2_ESM.pdf]

Supplementary File 2: Full dyad-level interaction results

This supplementary file provides the full dyad-level interaction results for the remaining 14 dyads and the complete FT1-ML4 results. Null-model panels follow the same layout as Figure 3, and combined interaction-index and home-range panels follow the same layout as Figure 4. Red stars in null-model panels denote significant deviations from the CRW null model at $p < 0.01$.

S2.1 Full FT1-ML4 results

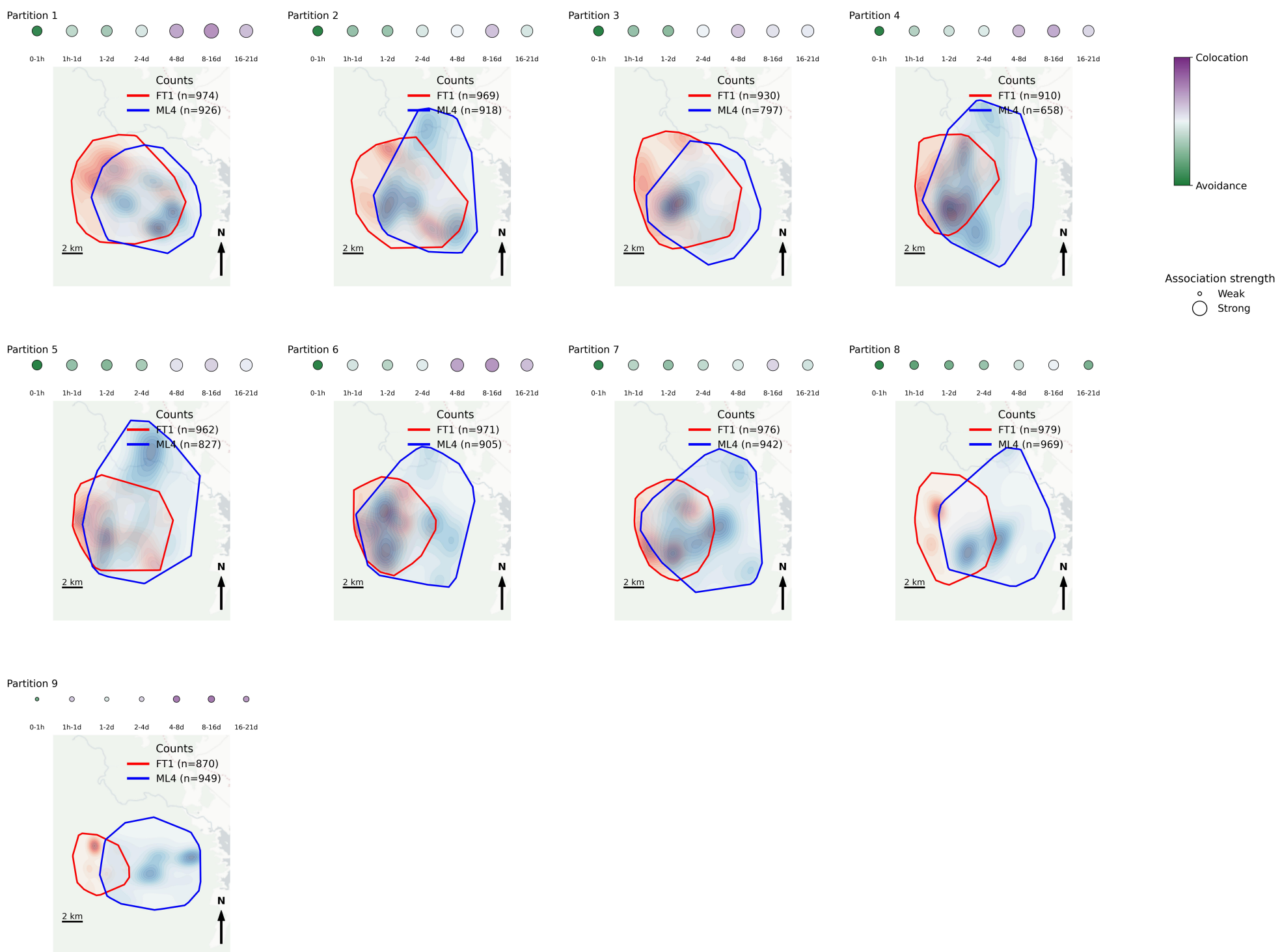

Fig. S1: Combined interaction-index and home-range visualization for FT1-ML4 across all valid partitions.

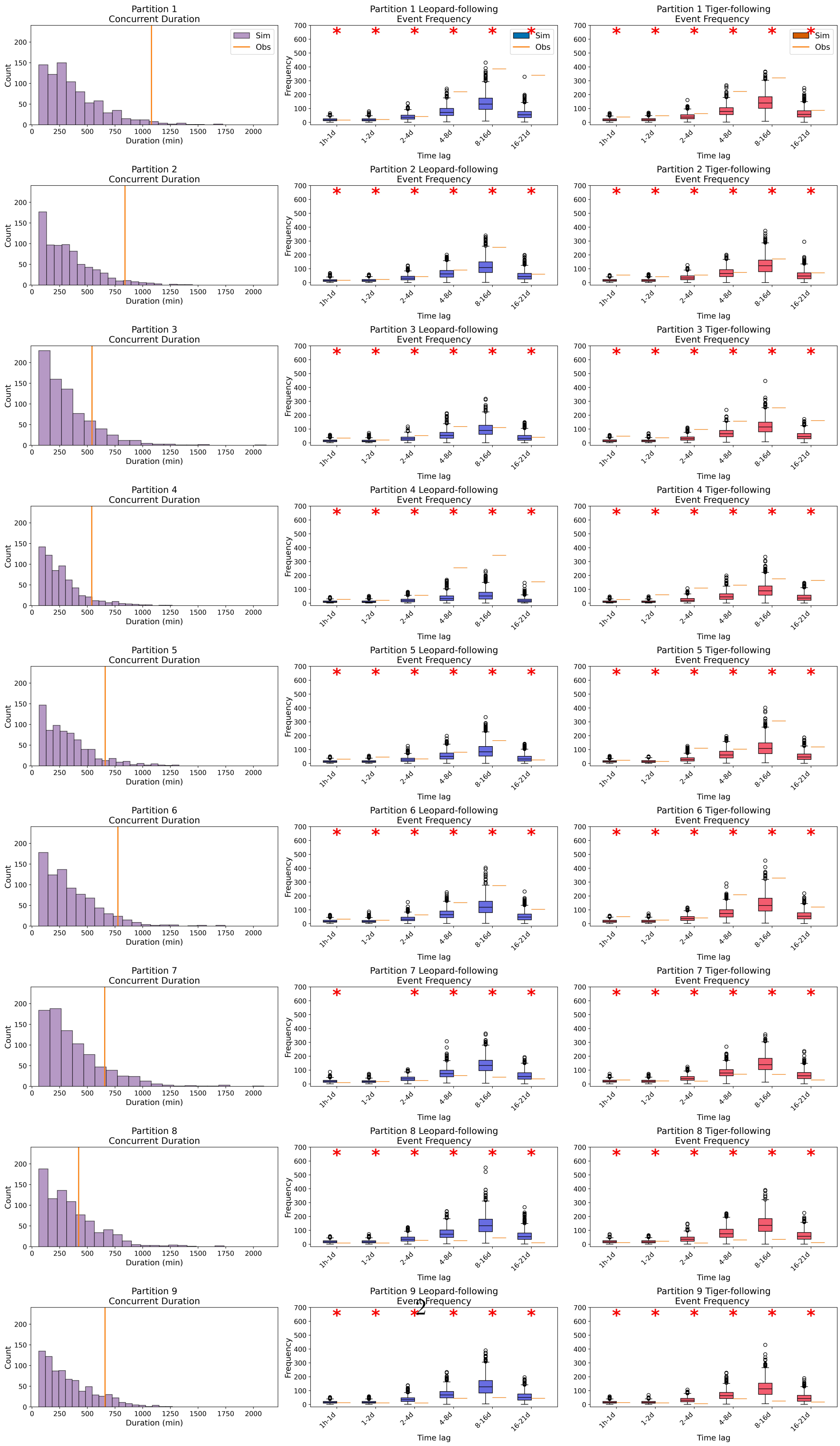

Fig. S2: Null-model interaction results for FT1-ML4 across all valid partitions.

S2.2 Home range shift competition dyads

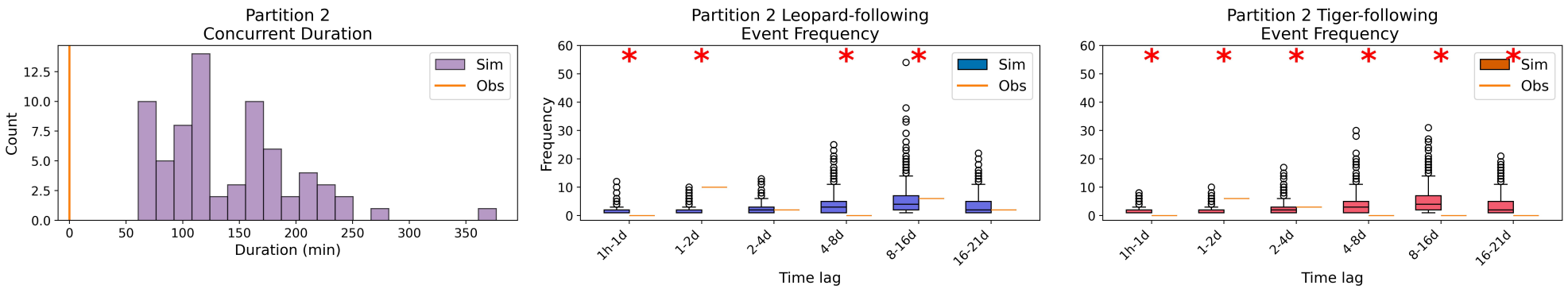

Fig. S3: Null-model interaction results for FT1-FL.

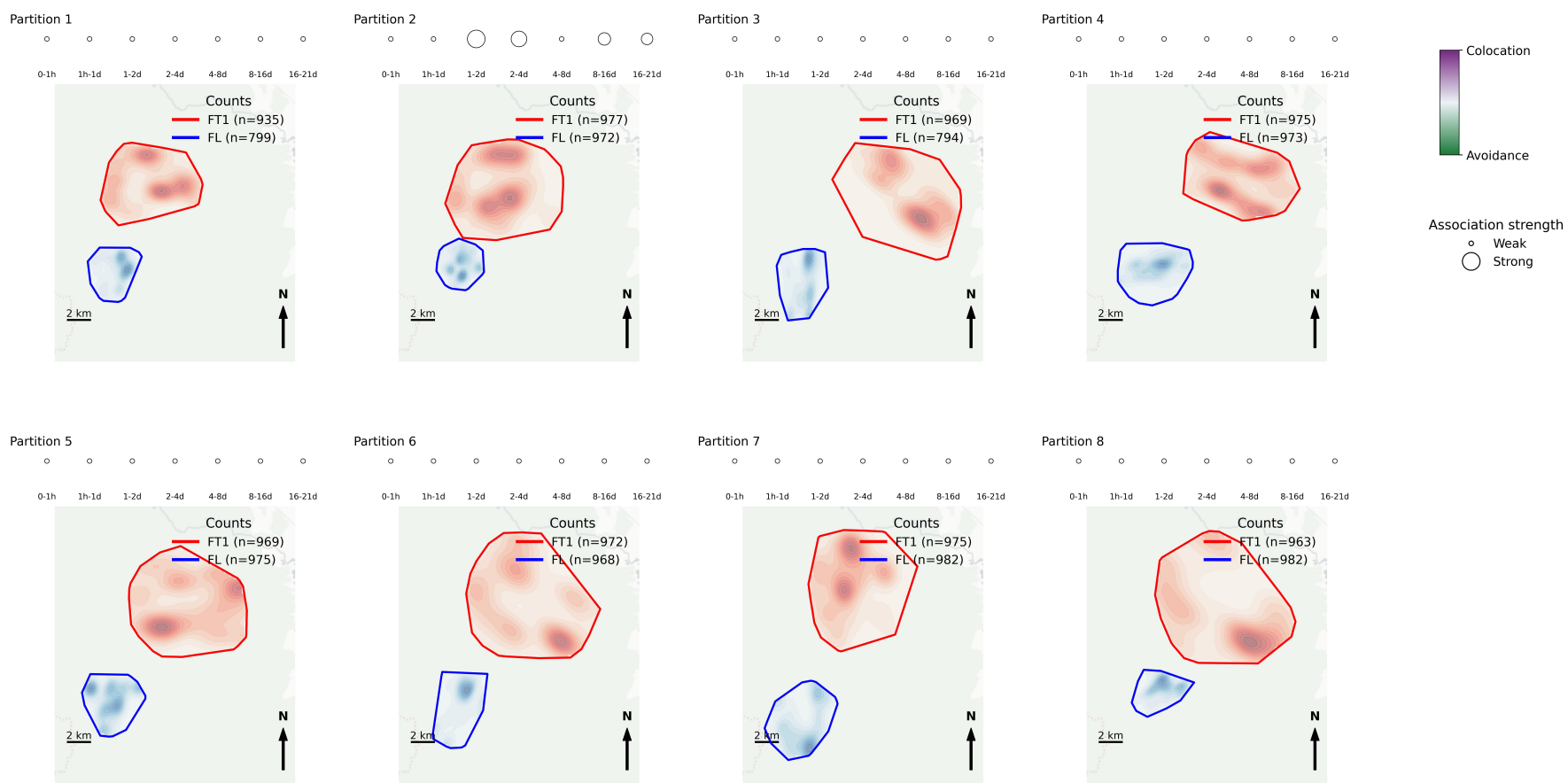

Fig. S4: Combined interaction-index and home-range visualization for FT1-FL.

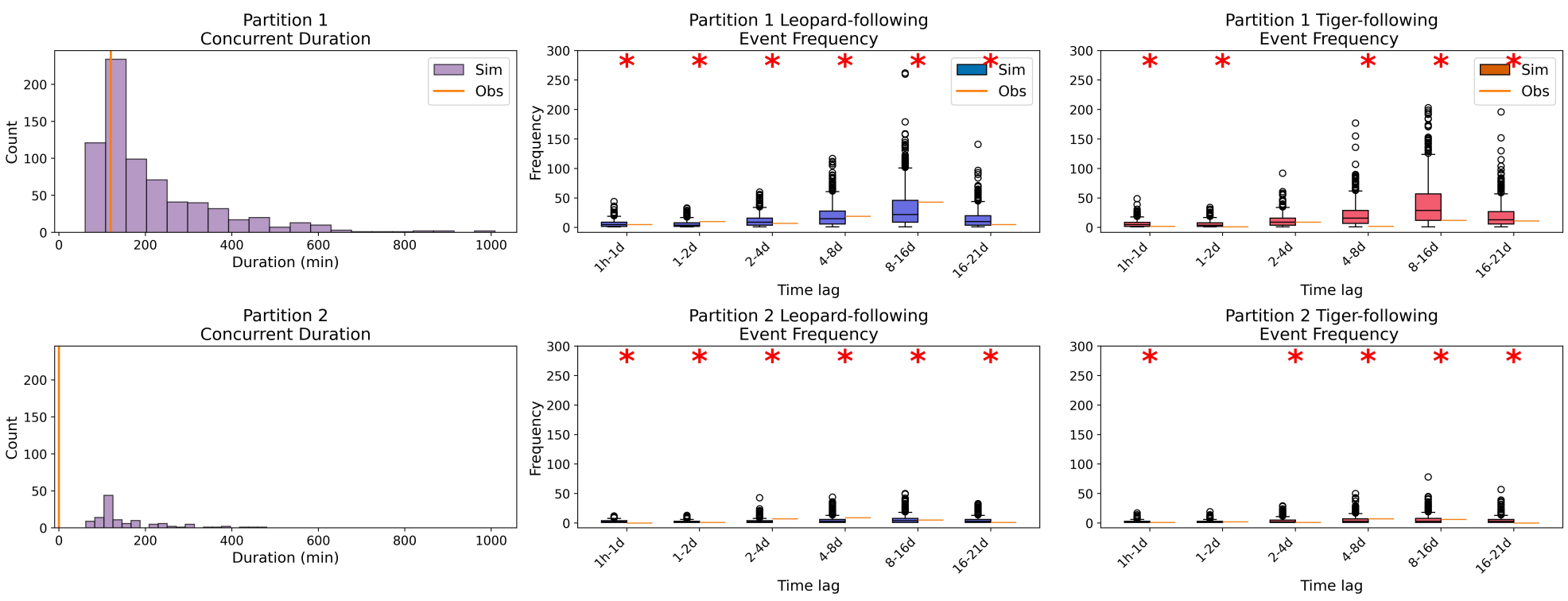

Fig. S5: Null-model interaction results for FT1-ML3.

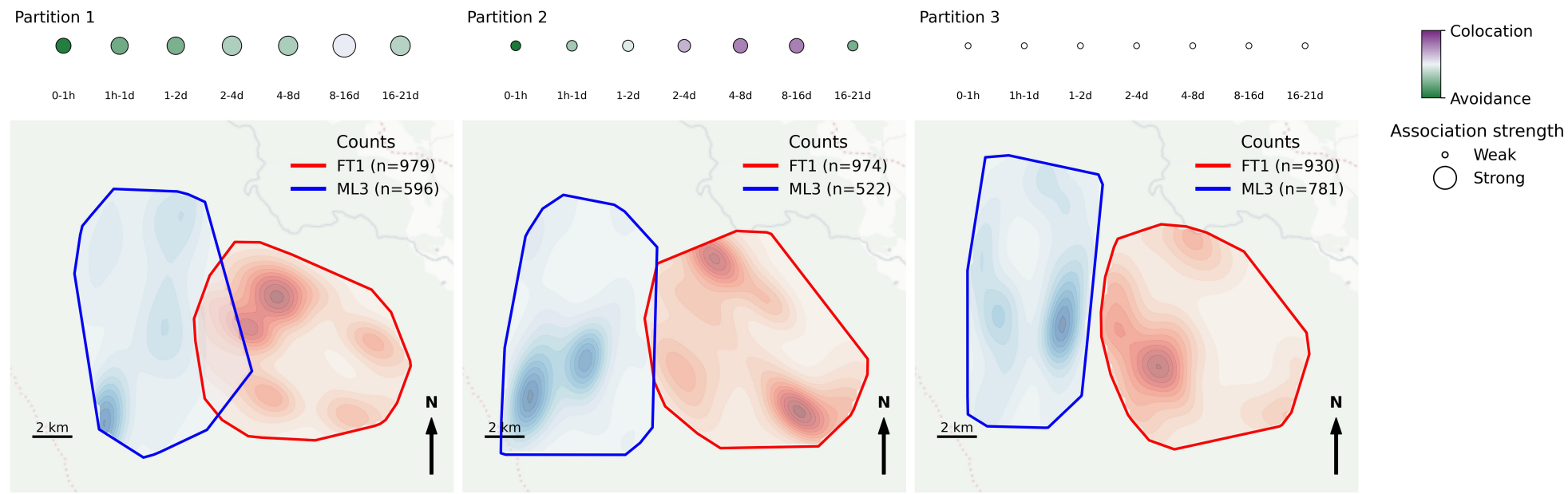

Fig. S6: Combined interaction-index and home-range visualization for FT1-ML3.

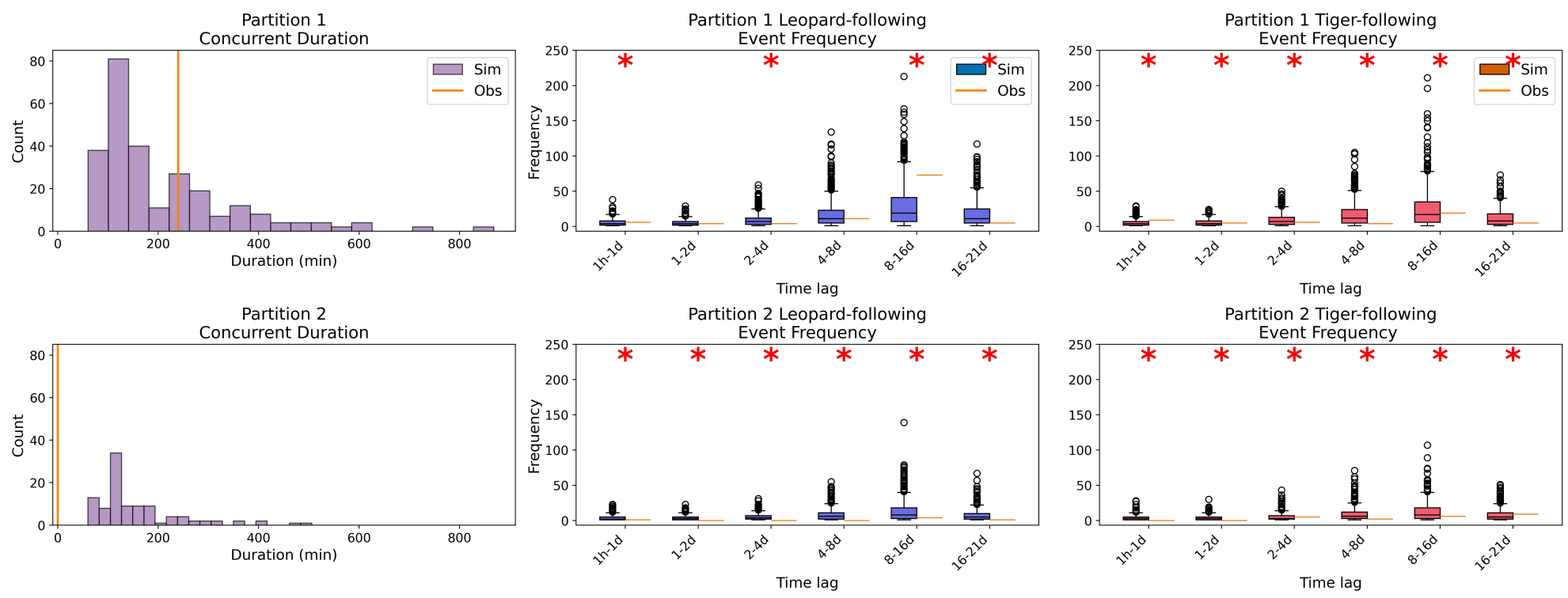

Fig. S7: Null-model interaction results for MT2-ML3.

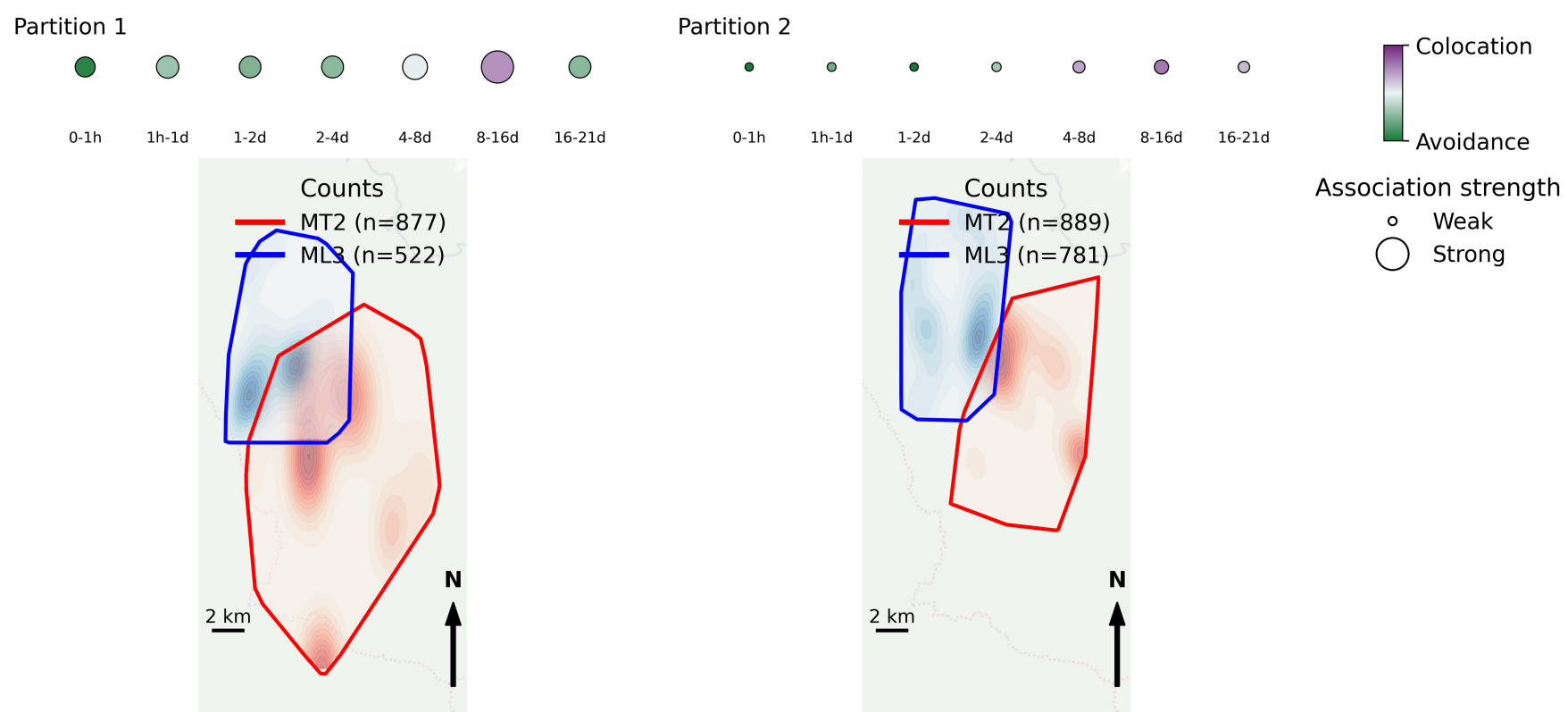

Fig. S8: Combined interaction-index and home-range visualization for MT2-ML3.

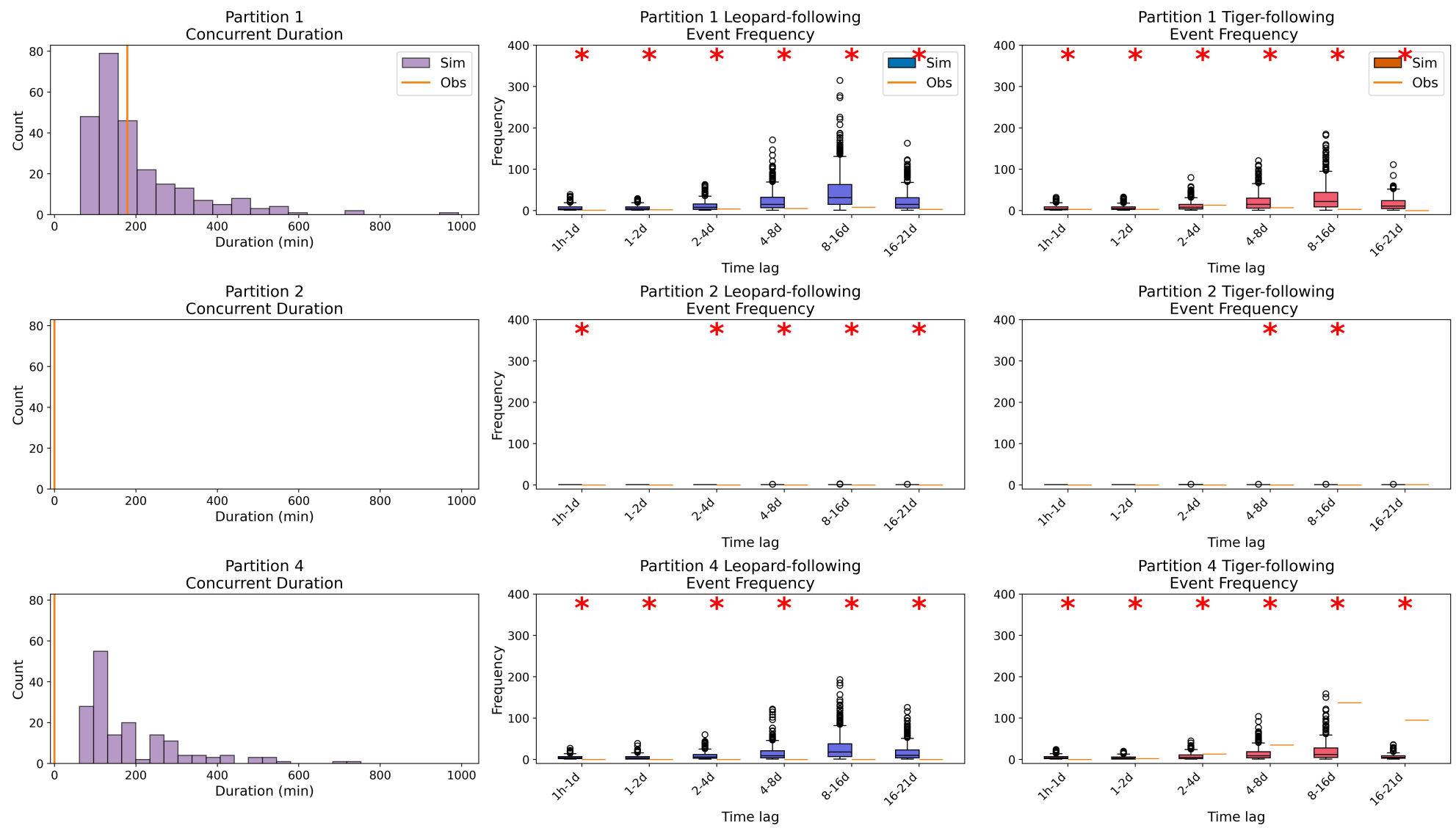

Fig. S9: Null-model interaction results for FT3-FL.

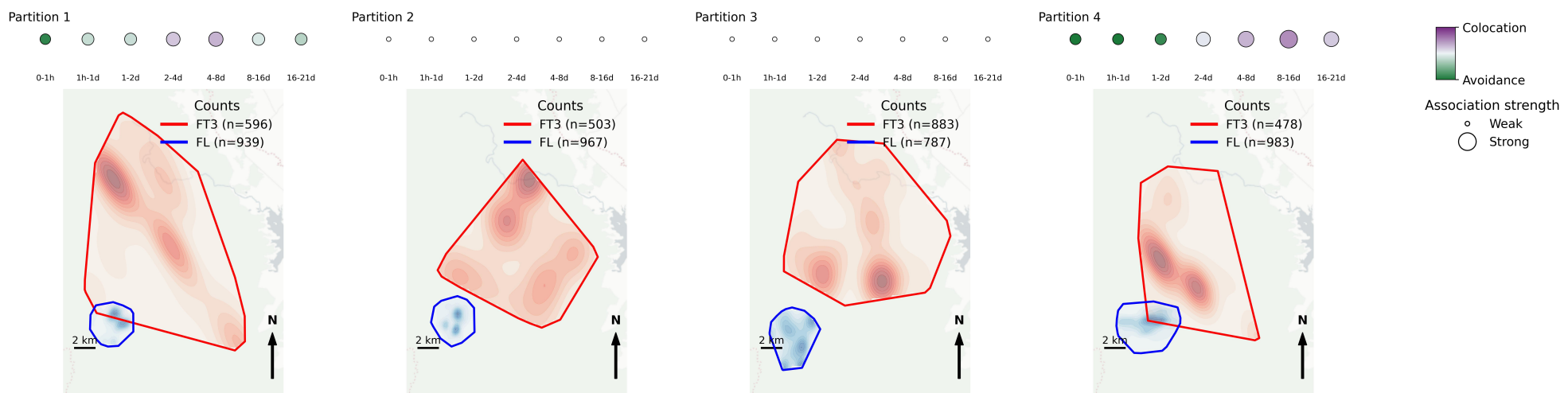

Fig. S10: Combined interaction-index and home-range visualization for FT3-FL.

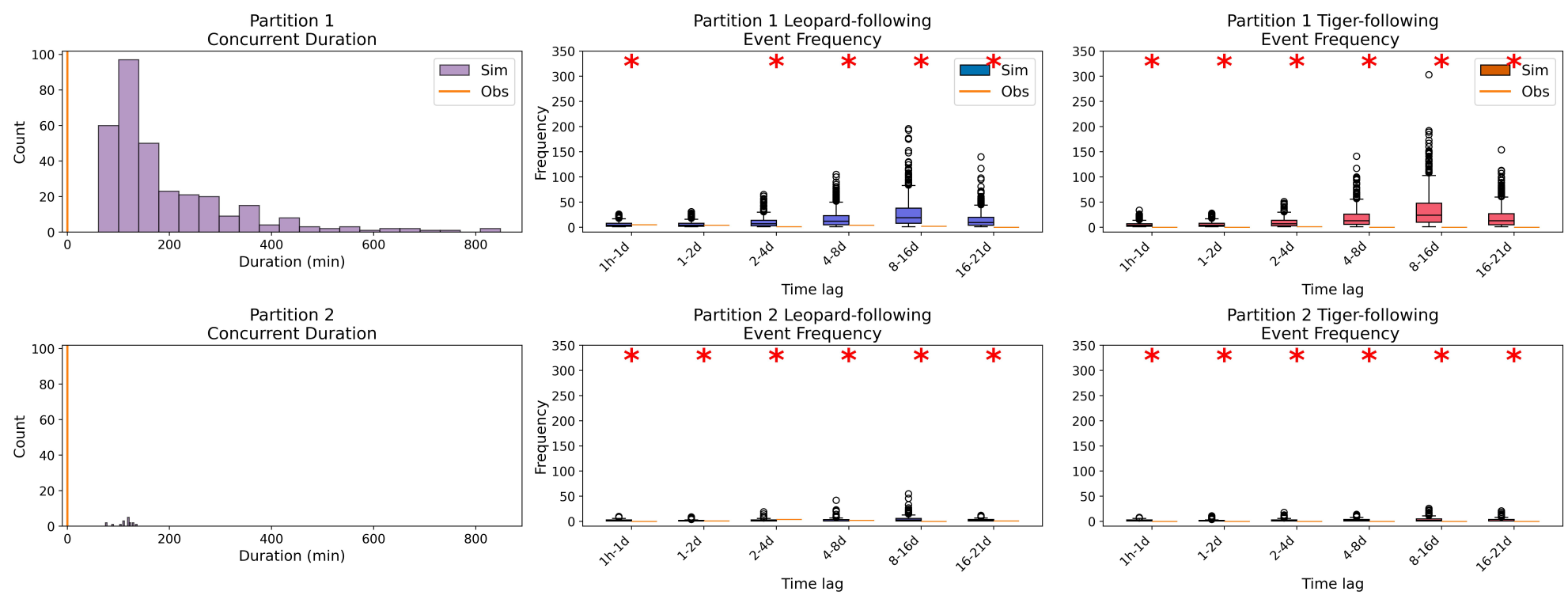

Fig. S11: Null-model interaction results for MT3-ML3.

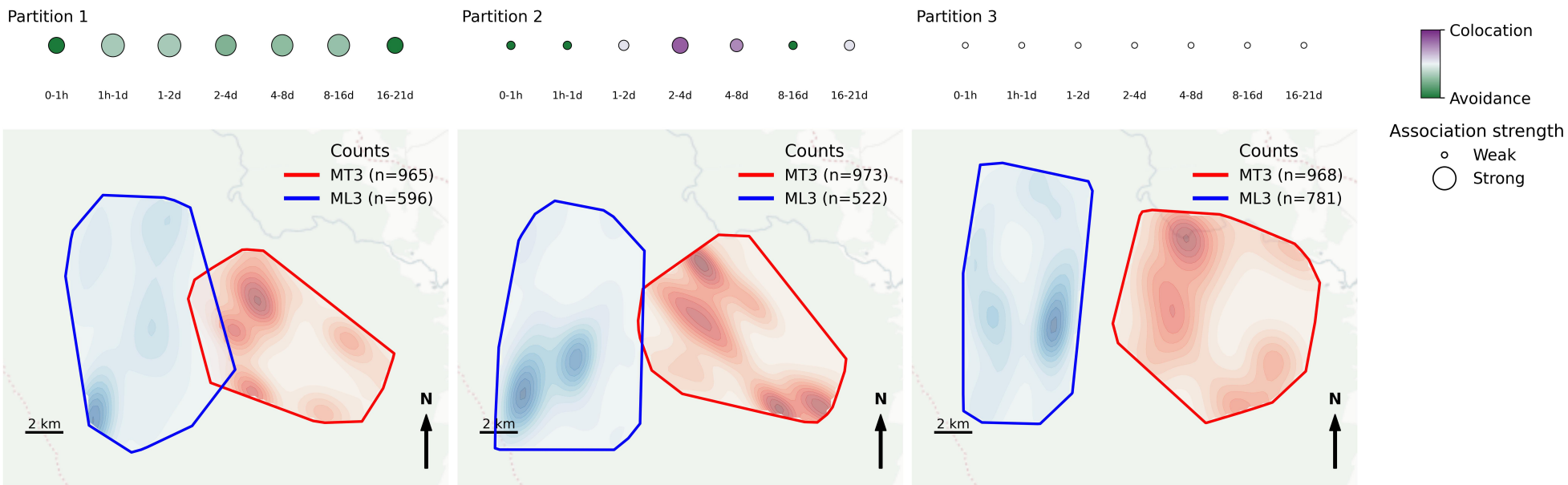

Fig. S12: Combined interaction-index and home-range visualization for MT3-ML3.

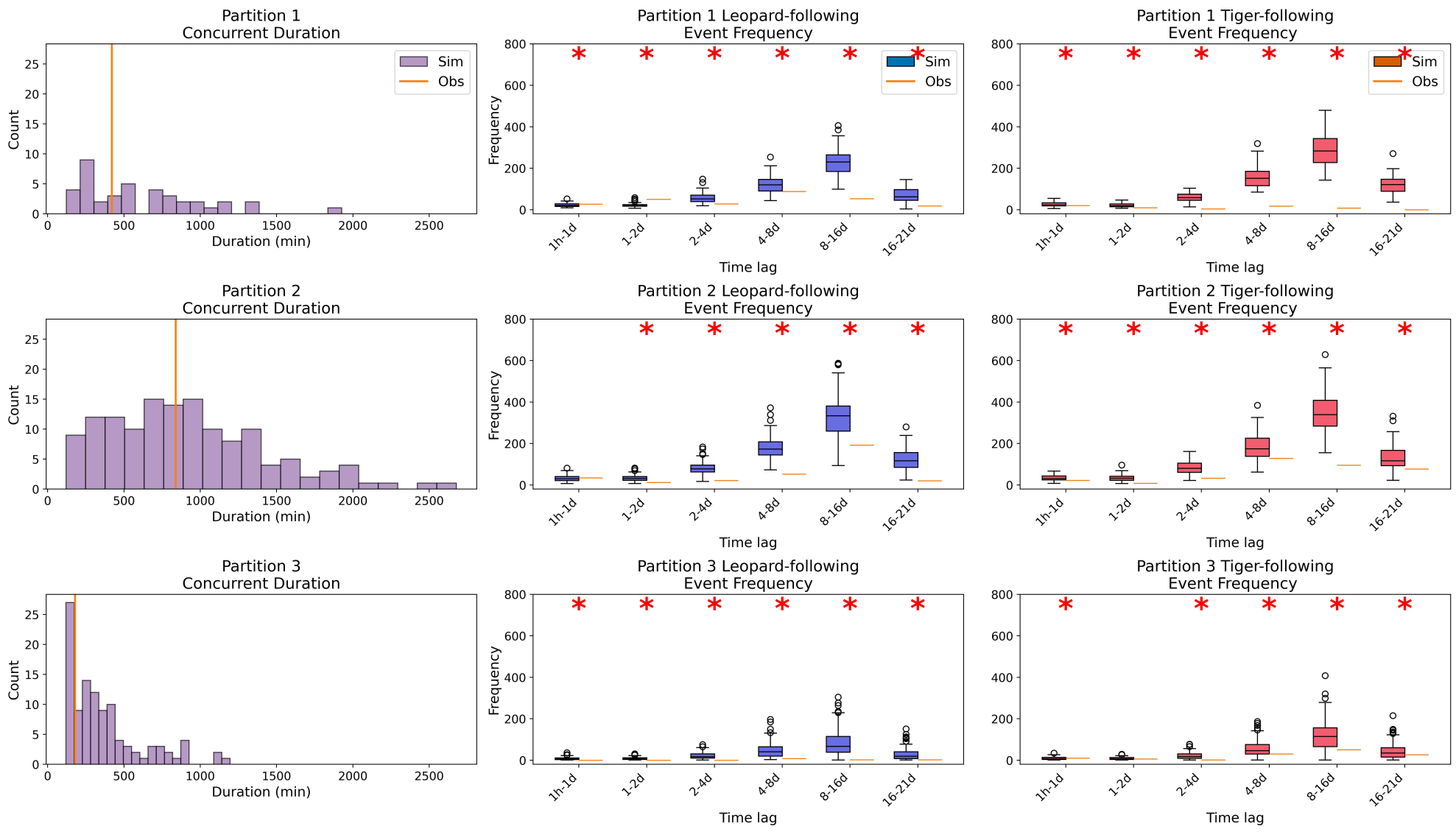

Fig. S13: Null-model interaction results for MT3-ML1.

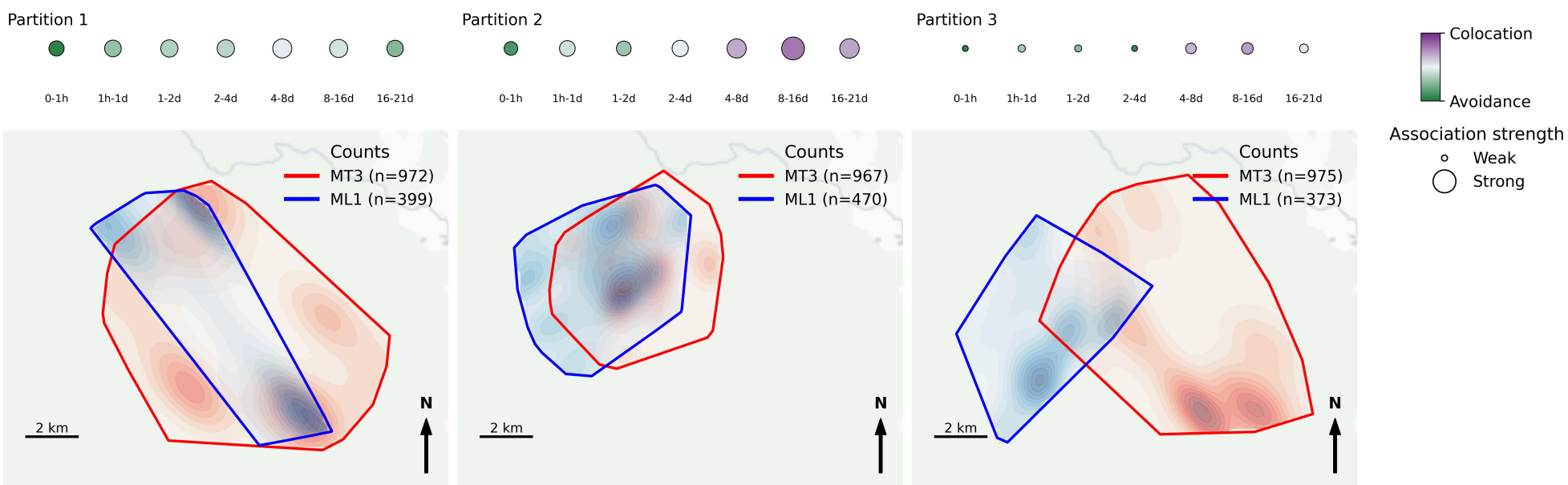

Fig. S14: Combined interaction-index and home-range visualization for MT3-ML1.

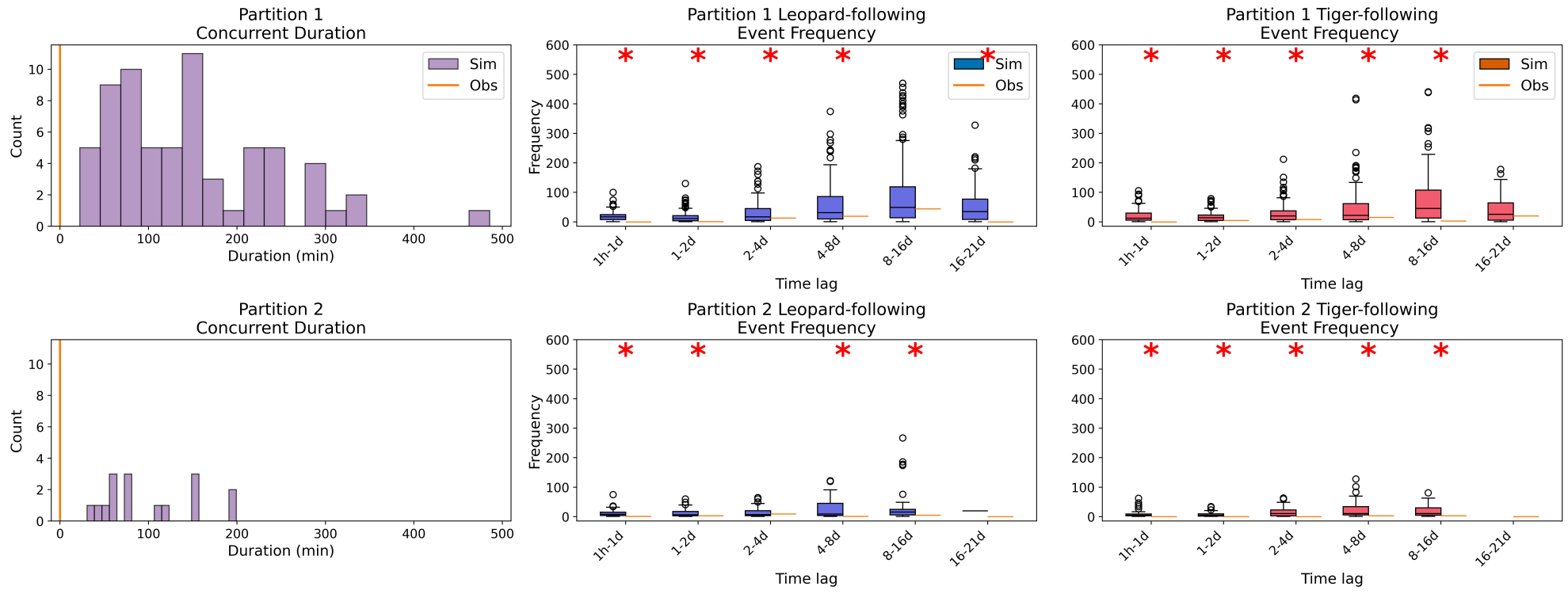

Fig. S15: Null-model interaction results for FT2-ML1.

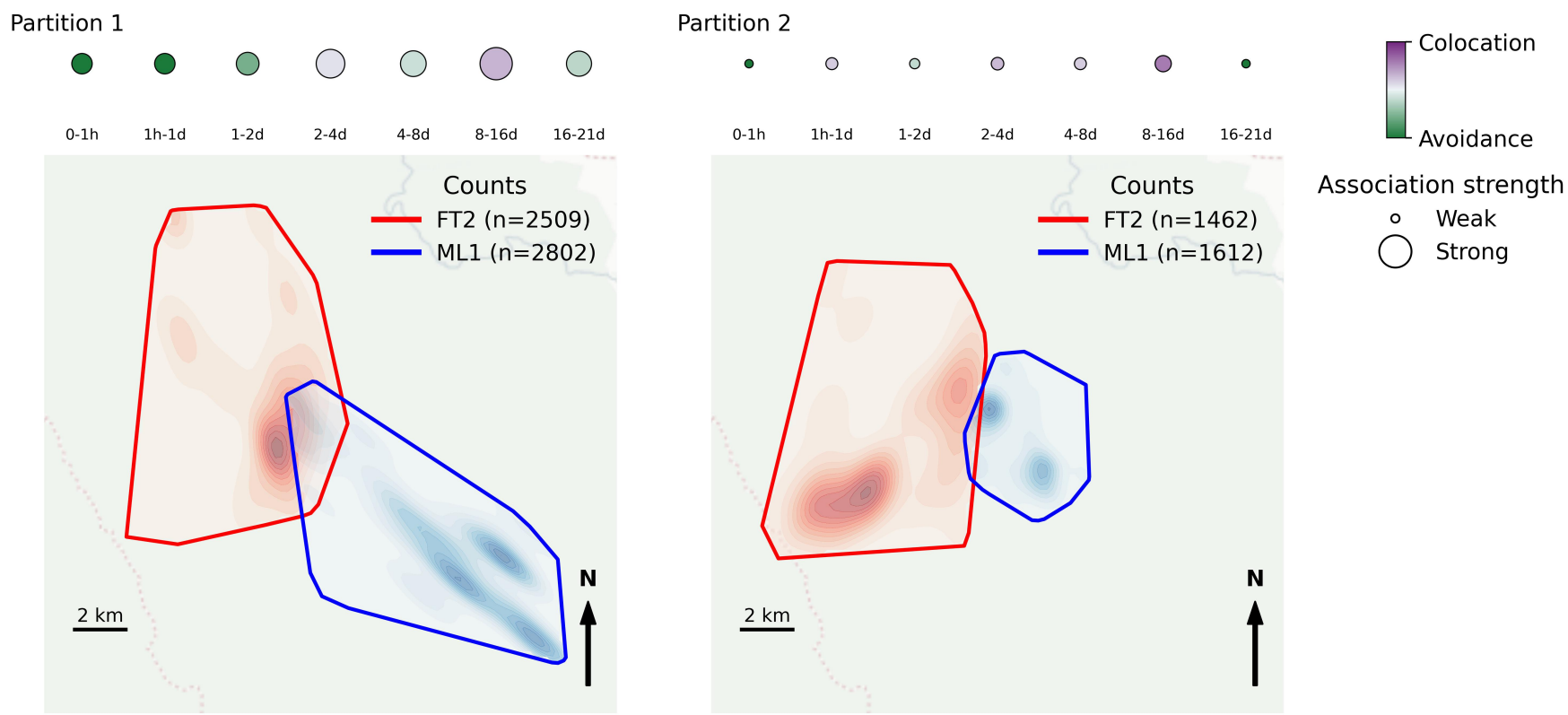

Fig. S16: Combined interaction-index and home-range visualization for FT2-ML1.

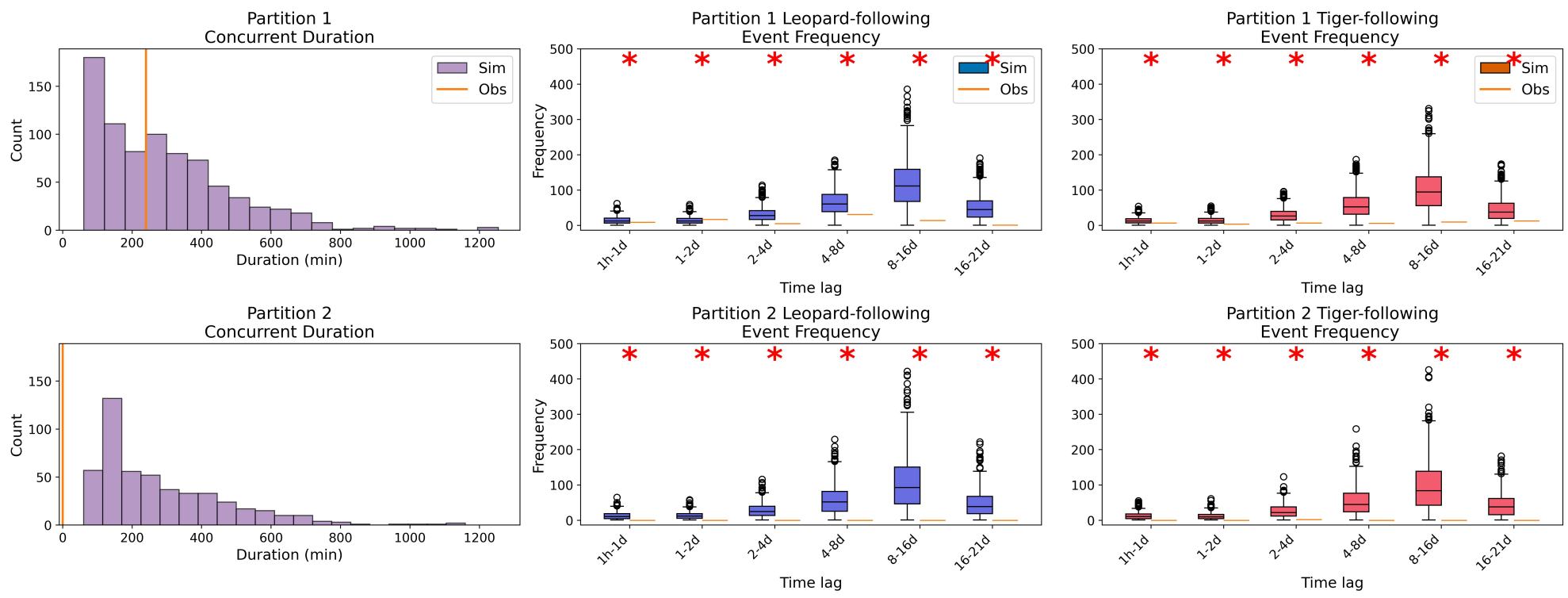

Fig. S17: Null-model interaction results for MT2-FL.

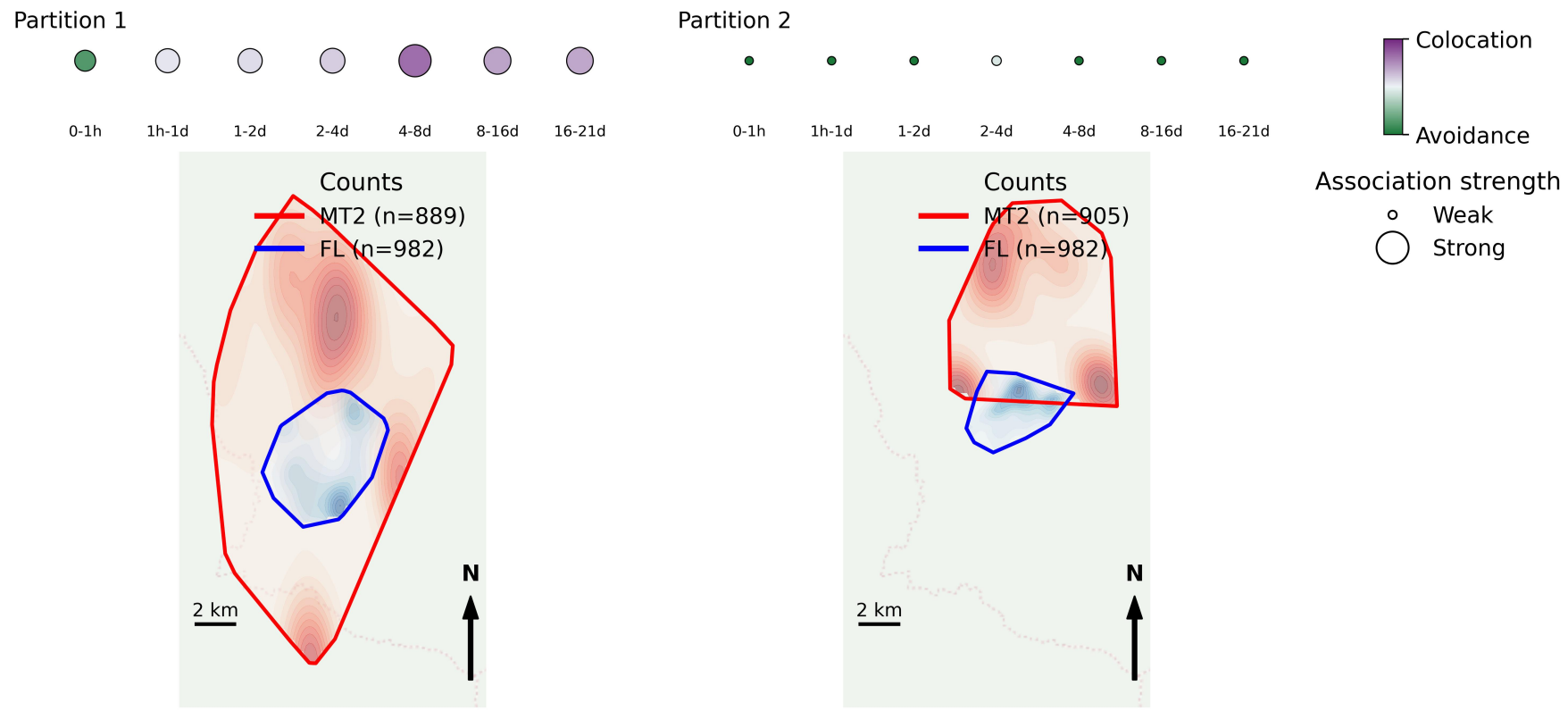

Fig. S18: Combined interaction-index and home-range visualization for MT2-FL.

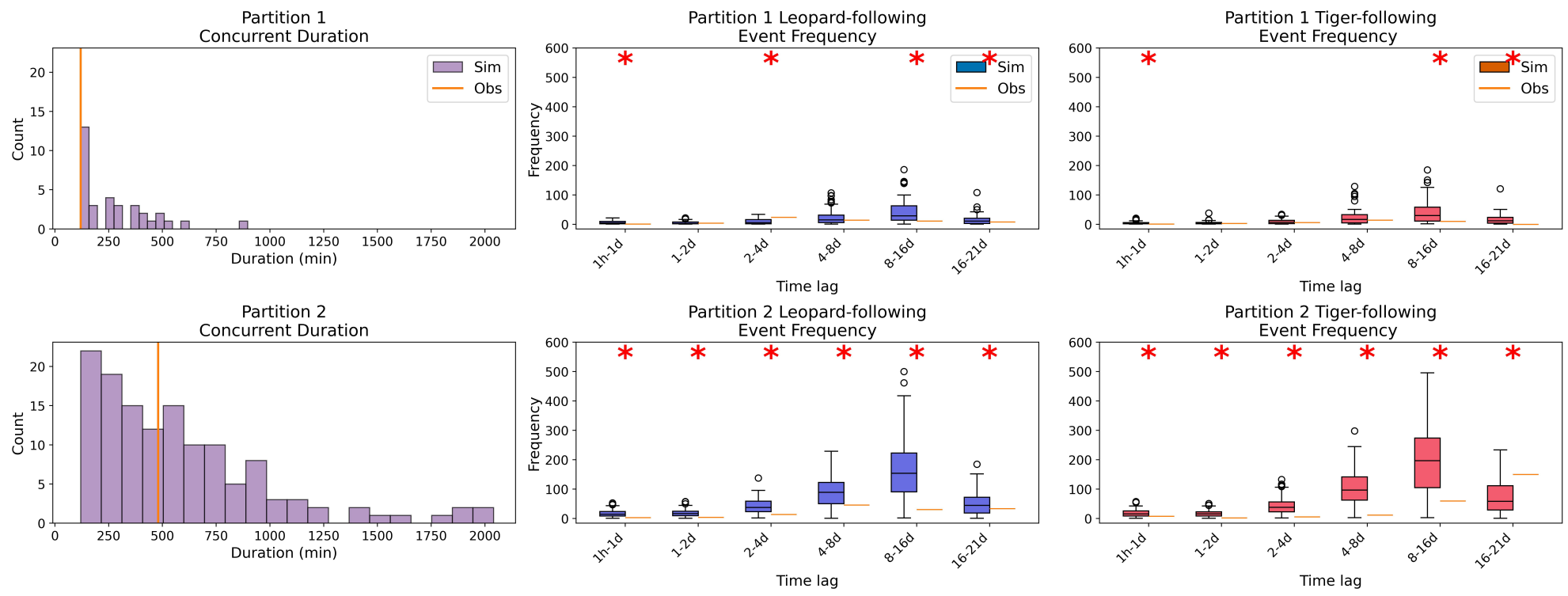

Fig. S19: Null-model interaction results for MT2-ML1.

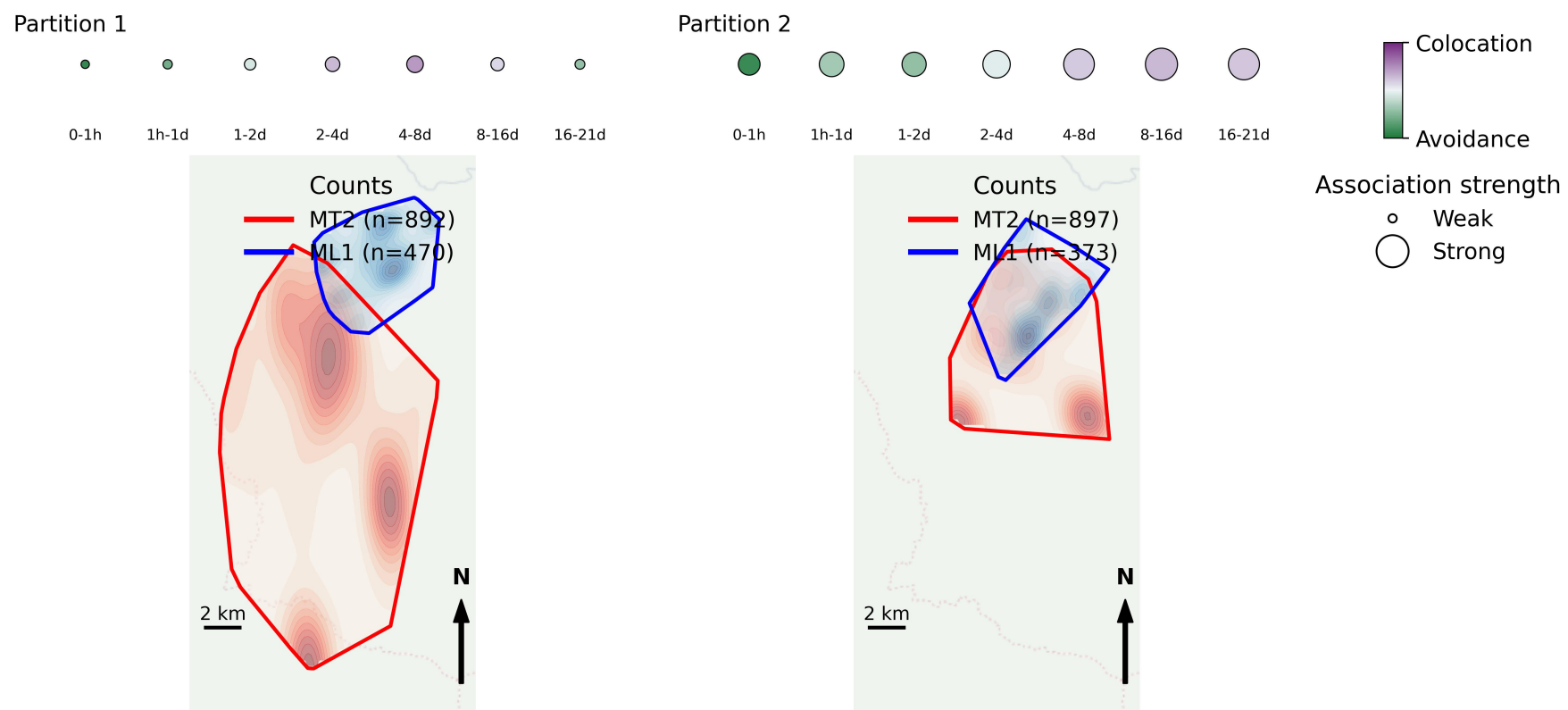

Fig. S20: Combined interaction-index and home-range visualization for MT2-ML1.

S2.3 Stable coexistence dyads

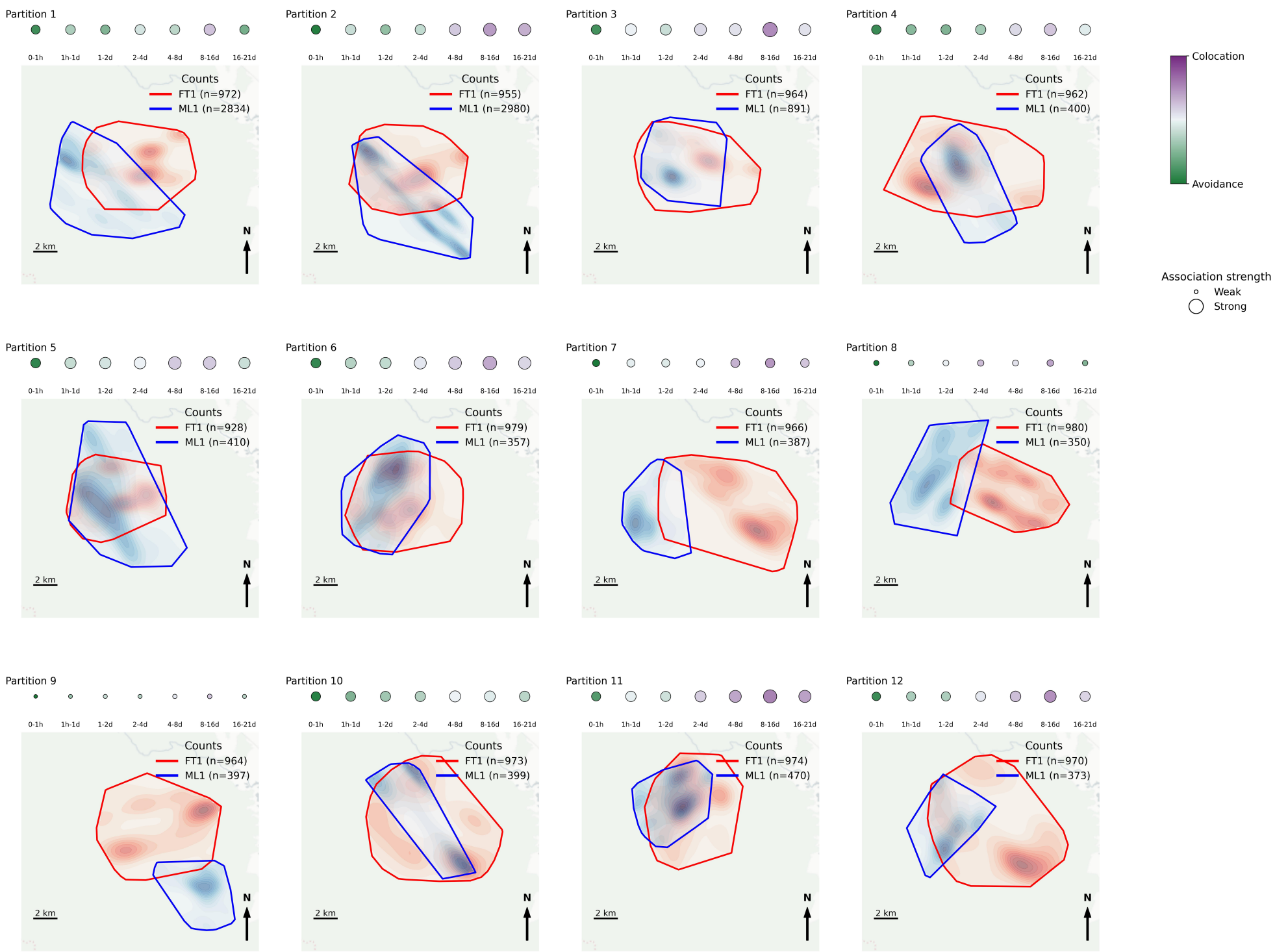

Fig. S21: Combined interaction-index and home-range visualization for FT1-ML1.

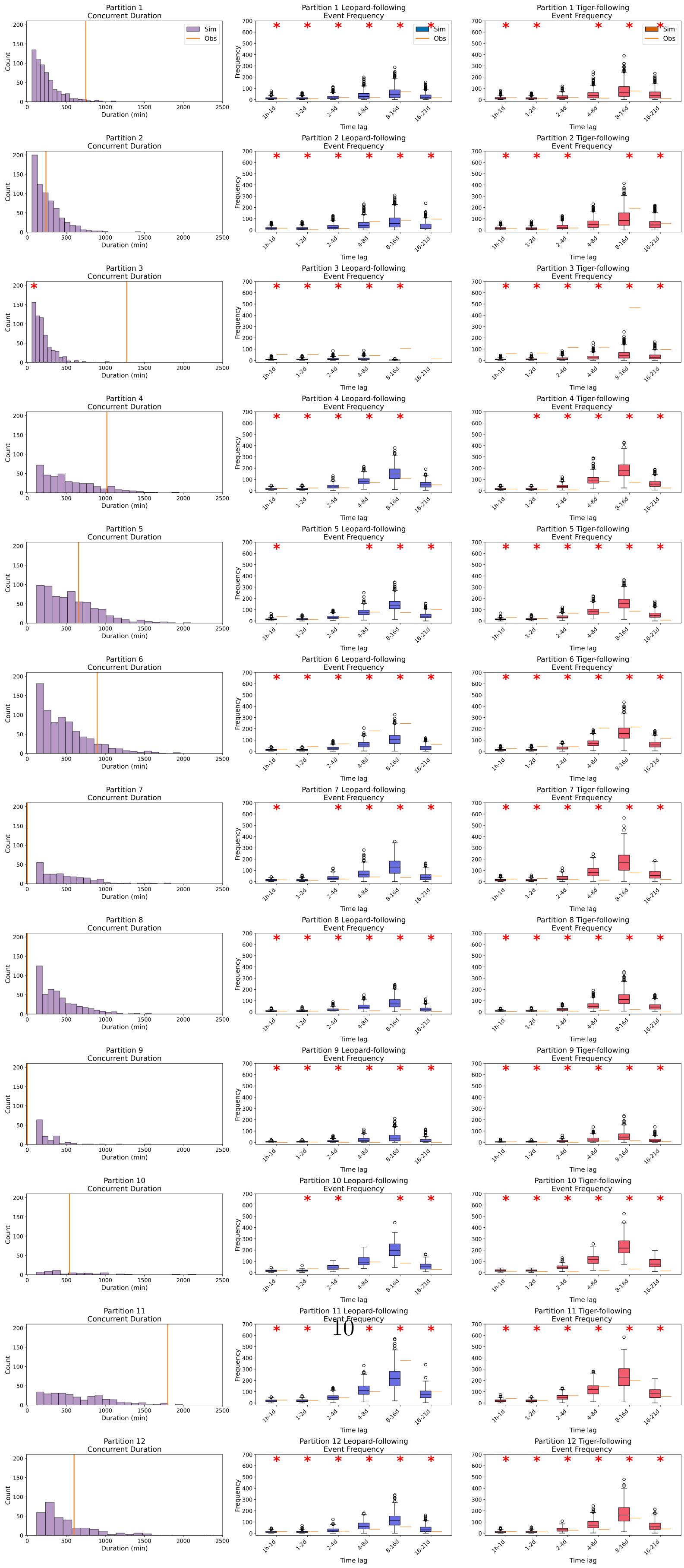

Fig. S22: Null-model interaction results for FT1-ML1.

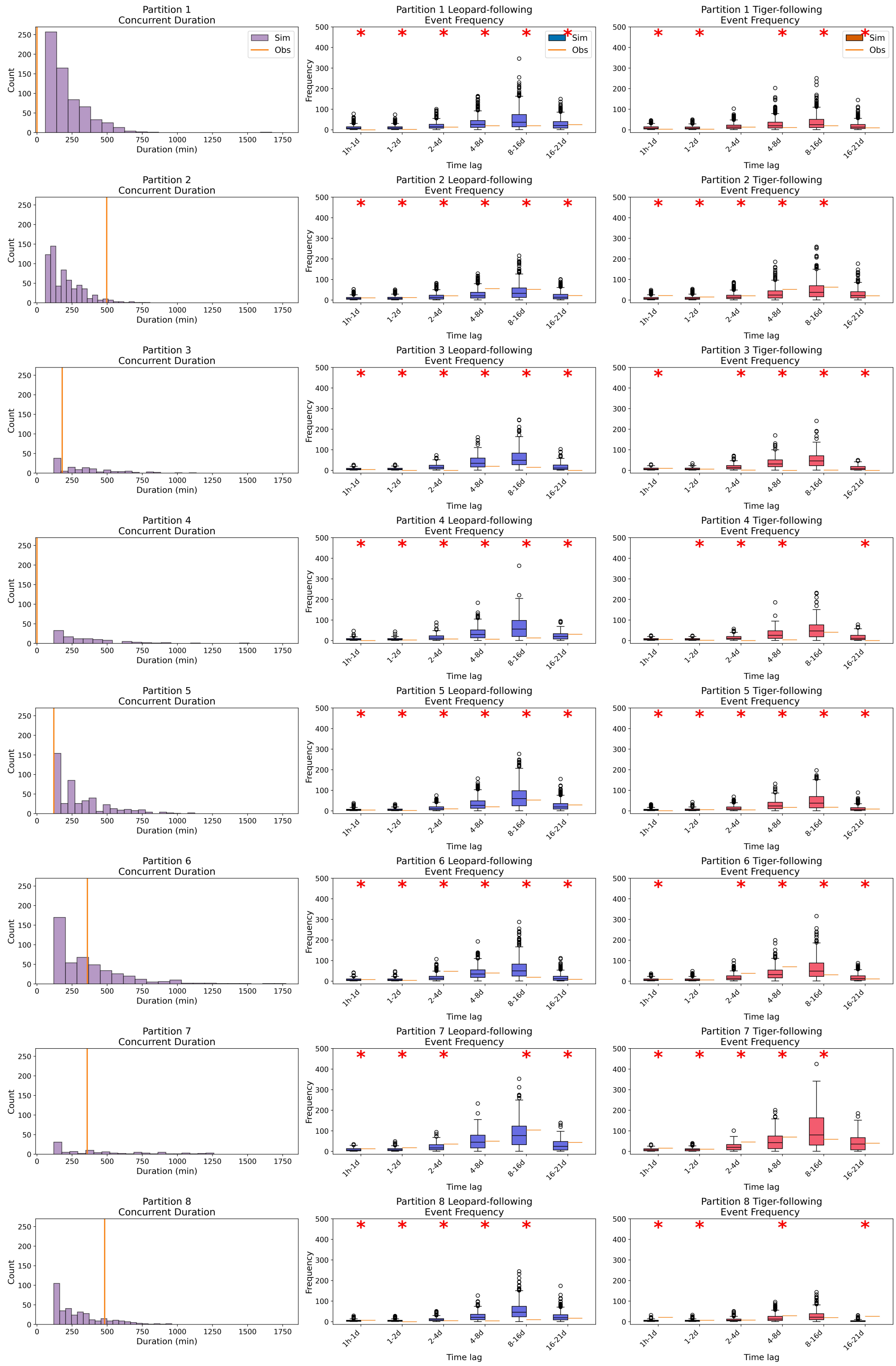

Fig. S23: Null-model interaction results for FT3-ML1.

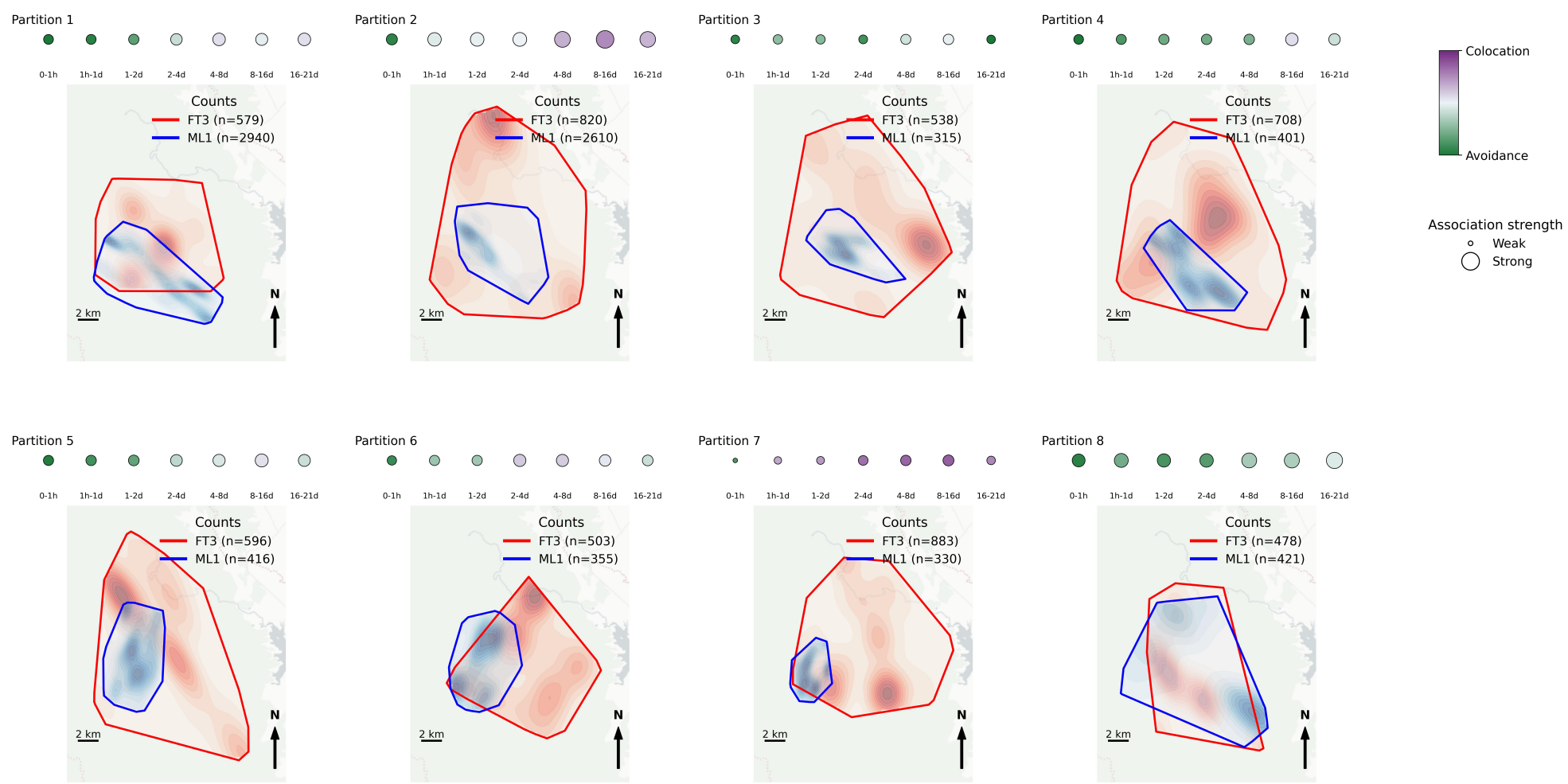

Fig. S24: Combined interaction-index and home-range visualization for FT3-ML1.

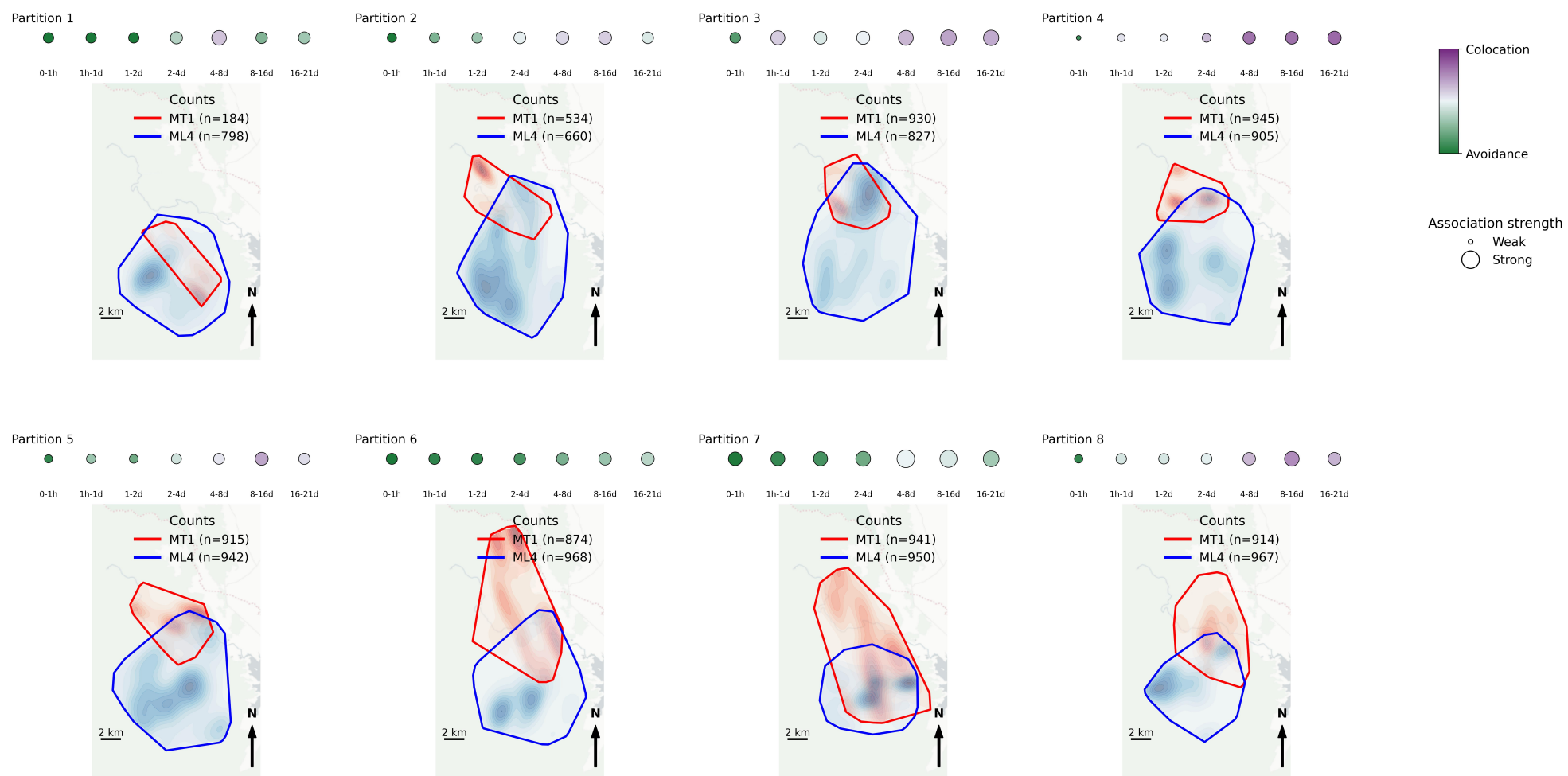

Fig. S25: Combined interaction-index and home-range visualization for MT1-ML4.

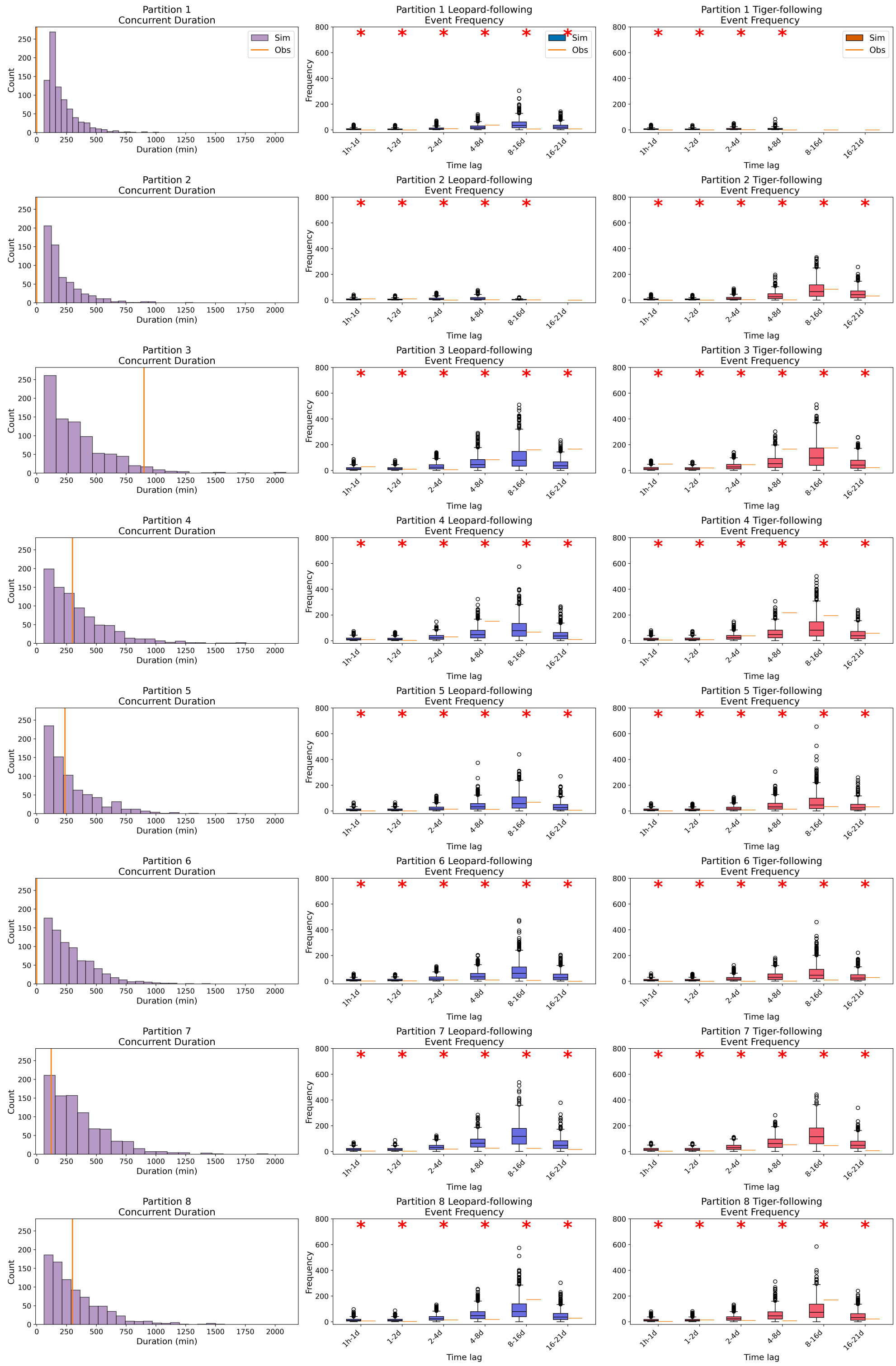

Fig. S26: Null-model interaction results for MT1-ML4.

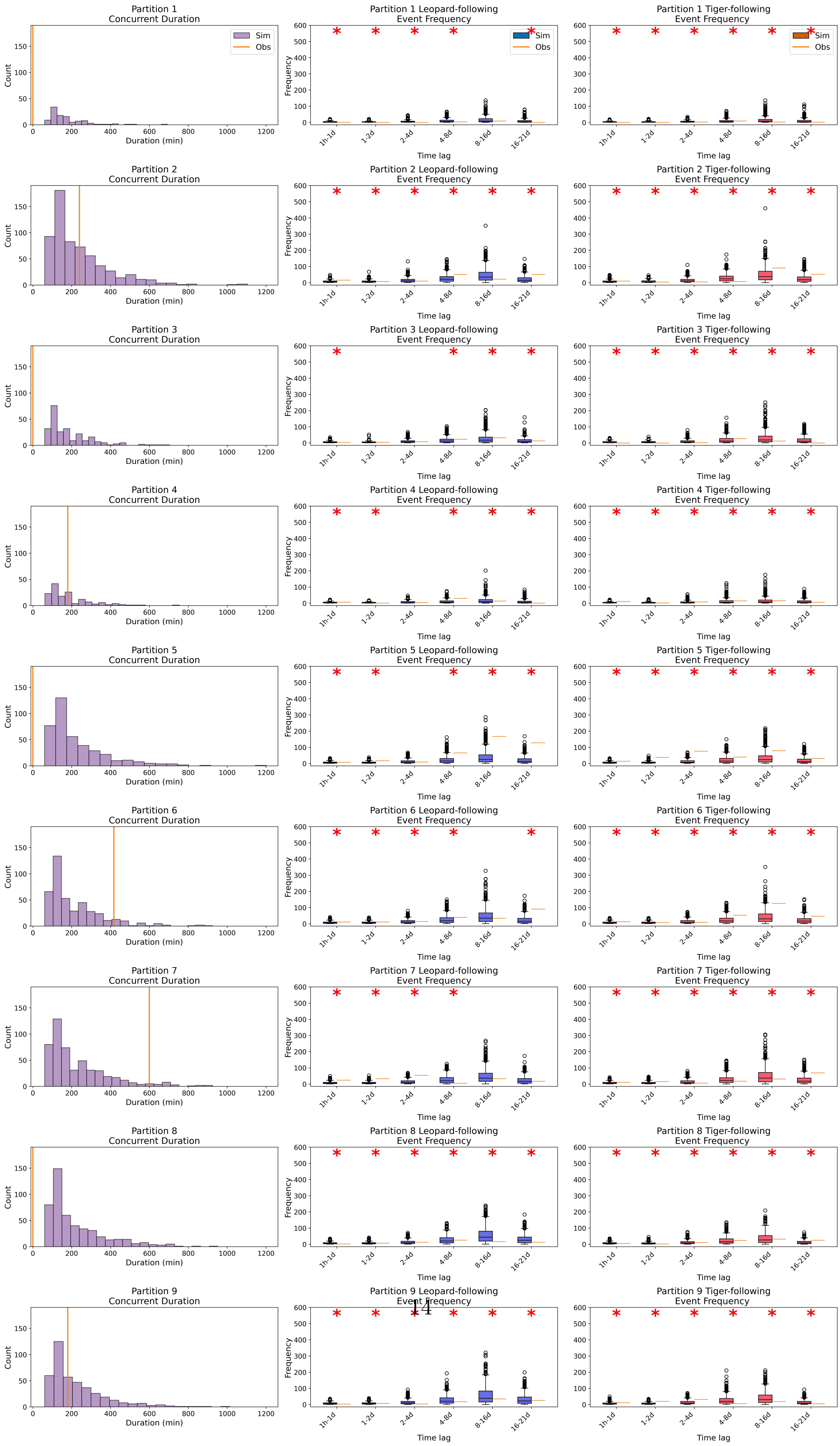

Fig. S27: Null-model interaction results for MT2-ML4.

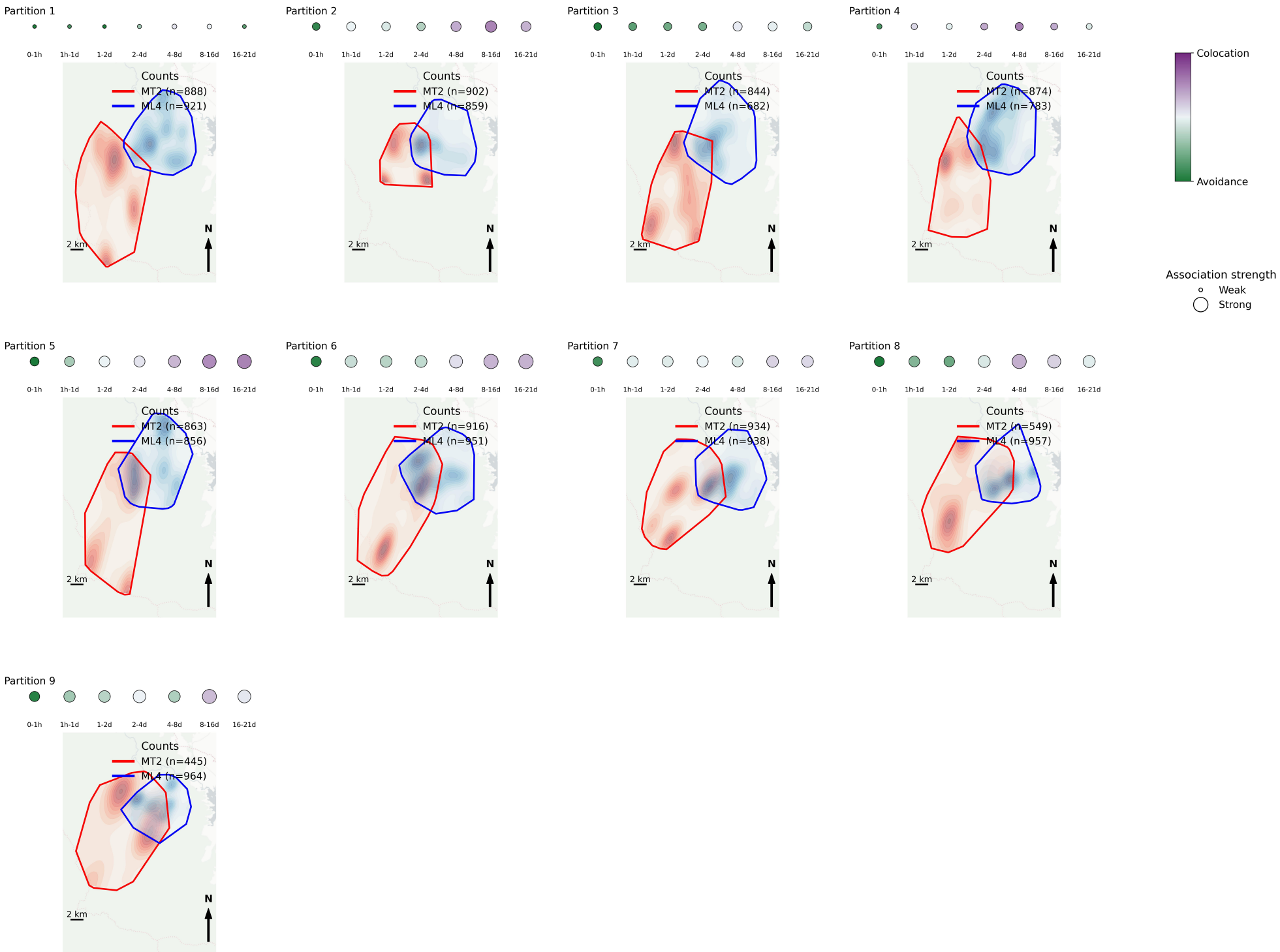

Fig. S28: Combined interaction-index and home-range visualization for MT2-ML4.

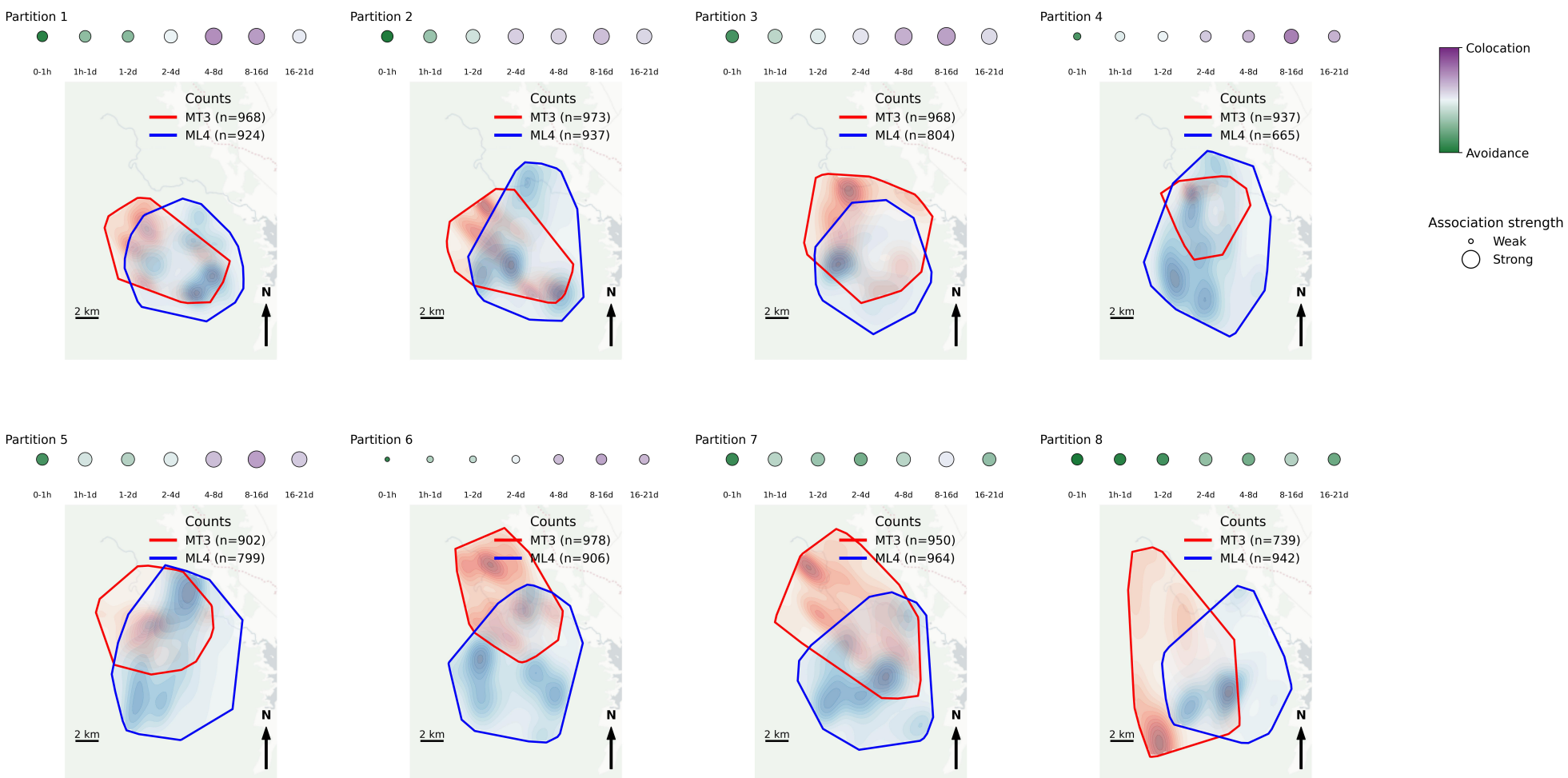

Fig. S29: Combined interaction-index and home-range visualization for MT3-ML4.

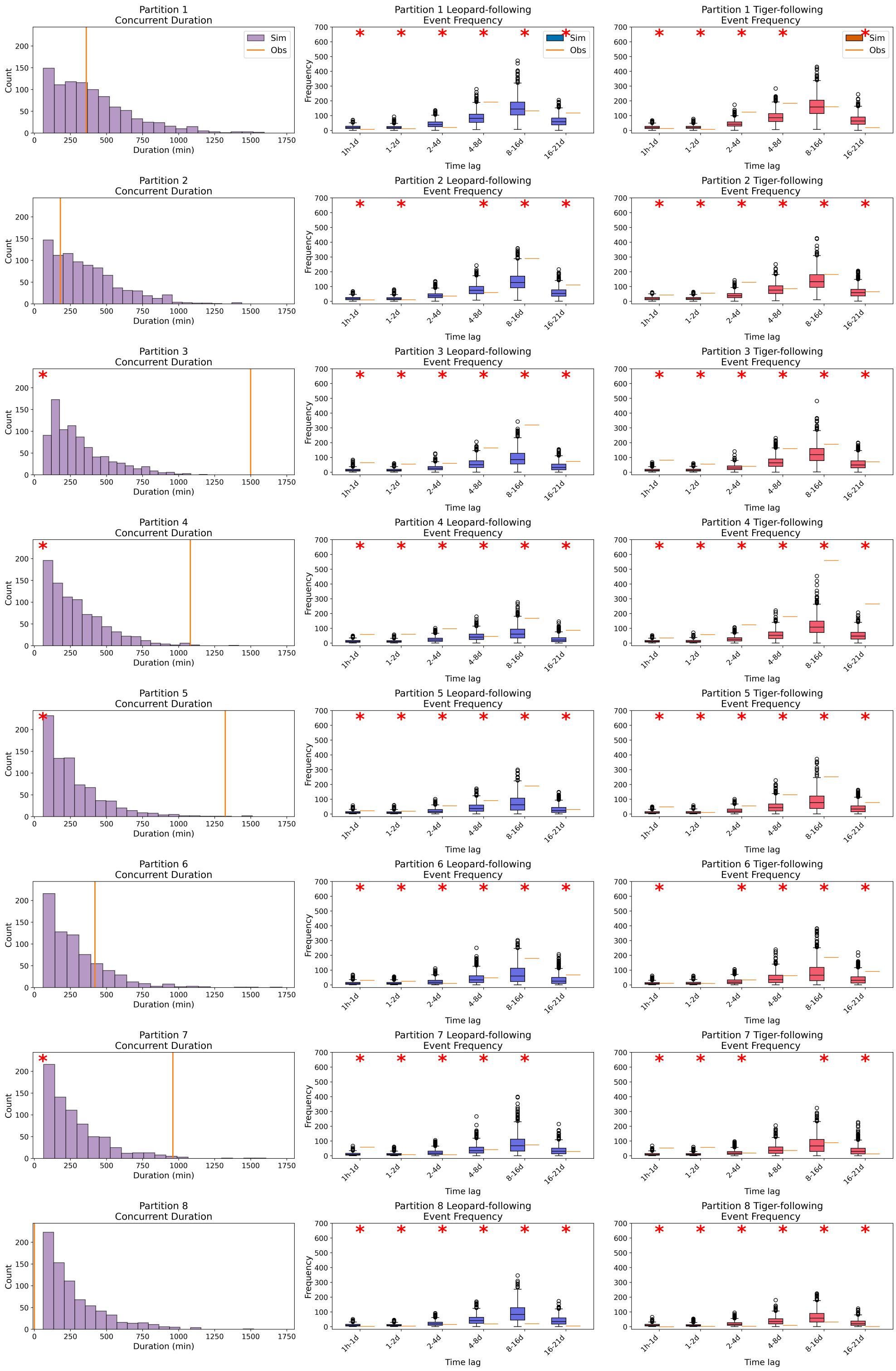

Fig. S30: Null-model interaction results for MT3-ML4.
